# Environmental DNA Epigenetics Accurately Predicts the Age of Cultured Fish Larvae

**DOI:** 10.1002/ece3.70645

**Published:** 2025-02-12

**Authors:** Eliot Ruiz, Fabien Leprieur, Gérard Sposito, Martina Lüthi, Michel Schmidlin, Jacques Panfili, Loïc Pellissier, Camille Albouy

**Affiliations:** ^1^ MARBEC, Univ Montpellier, IRD, IFREMER, CNRS Montpellier France; ^2^ Mediterranean Coastal Environment Station University of Montpellier Sète France; ^3^ Department of Environmental Systems Science, Ecosystems and Landscape Evolution, Institute of Terrestrial Ecosystems ETH Zürich Zürich Switzerland; ^4^ Land Change Science Research Unit Swiss Federal Research Institute WSL Birmensdorf Switzerland

**Keywords:** age estimation, environmental DNA, epigenetic clock, fish larvae, methylation, nanopore sequencing

## Abstract

While acquiring age information is crucial for efficient stock management and biodiversity conservation, traditional aging methods fail to offer a universal, non‐invasive, and precise way of estimating a wild animal's age. DNA methylation from tissue DNA (tDNA) was recently proposed as a method to overcome these issues and showed more accurate results than telomere‐length‐based age assessments. Here, we used environmental DNA (eDNA) for the first time as a template for age estimation, focusing on the larval phase (10–24 days post‐hatch) of cultured *Dicentrarchus labrax* (seabass), a species of major economic and conservation interest. Using third‐generation sequencing, we were able to directly detect various modification types (e.g., cytosine and adenosine methylation in all contexts) across the whole genome using amplification‐free nanopore sequencing. However, aging sites were only present in the mitogenome, which could be a specific feature of eDNA methylation or the consequence of better DNA protection within mitochondria. By considering qualitative and quantitative information about aging sites according to an objective model selection framework, our epigenetic clock reached a cross‐validated accuracy of 2.6 days (Median Absolute Error). Such performances are higher than those of previous clocks, notably for adult seabass even when scaling MAE to the age range, which could be linked to a more dynamic epigenome during early life stages. Overall, our pilot study proposes new methods to determine the potential of eDNA for simultaneous age and biodiversity assessments, although robust validation of our preliminary results along with methodological developments are needed before field applications can be envisaged.

## Graphical abstract

While various biases generally hamper age estimation methods and are not universally applicable to all animal taxa, epigenetic patterns—in particular DNA methylation—were recently demonstrated to have the potential to revolutionize this field as they are accurate and standardized. In this manuscript, we present the first investigation into the potential of DNA methylation retrieved from environmental DNA (eDNA) for age prediction using epigenetic clocks. Our findings suggest that it is possible to predict the age of seabass larvae with a precision of 2.6 days based on mitogenomic aging sites, which could facilitate the non‐invasive study of age structures at the scale of whole ecosystems, capitalizing on the benefits of both eDNA sampling and epigenetic aging.

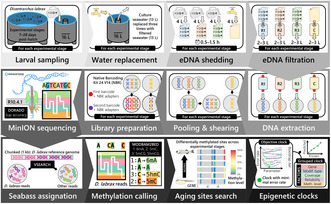

## 1 Introduction

Among the biological variables characterizing a population of wild animals, the age of individuals is one of the most relevant to acquire (Le Clercq et al. 2023; Piferrer and Anastasiadi 2023). Indeed, age assessments are carried out for a wide variety of purposes; for example, assessments of age‐class structures over multiple years are becoming crucial for the management of exploited populations and the conservation of threatened species (Piferrer and Anastasiadi 2023). Age‐based information, such as the duration of life stages (e.g., dispersal phase and reproductive maturity) or growth and mortality rates, is fundamental to parametrize both demographic (Iannelli and Milner 2017) and dispersal models (Swearer, Treml, and Shima 2019). These models allow conservation efforts to be prioritized toward the most vulnerable zones (e.g., major “source” areas, nursery zones) and stages (e.g., early juveniles and most fecund adults; Beger et al. 2022). Various complementary methods have been developed to assess the age of wild individuals based on size, mark‐recapture approaches (Amstrup, McDonald, and Manly 2005), or predictably evolving biochemical/morphological features (e.g., sclerochronology, pigments, and hormones; Zhang et al. 2024). However, they all have drawbacks in terms of resolution, universality (most are group‐specific), feasibility, efficiency, or cost (Le Clercq et al. 2023). To address these challenges, several genetic‐based methods have been proposed recently, such as telomere length (Haussmann and Vleck 2002). Cooke and Smith (1986) initially identified telomere length as a genetic feature that evolves with age in humans, and it has been used since 2002 to age more than 100 species (Le Clercq et al. 2023). However, a relationship between epigenetics and age was discovered more recently by Horvath (2013). Such a relationship has already been characterized for nearly 100 species, and epigenetics now appears to be more promising than telomere length for estimating age, in terms of resolution (Le Clercq et al. 2023).

Among the epigenetic mechanisms that control gene expression without actual changes to DNA sequences (e.g., histone/chromatin modification and non‐coding RNA), the potential of DNA methylation for constructing epigenetic clocks has been the most extensively studied to date (Booth and Brunet 2016; Trautner et al. 2017). DNA methylation is a reversible dynamic process that involves binding a methyl group or its oxidative derivatives (e.g., hydroxymethyl, formyl, or carboxyl group) to all four types of nucleotides (Carell et al. 2018; O'Brown and Greer 2016). Unlike epigenetic drift and environmental regulation, which are sources of variability between individuals' epigenomes, DNA methylation consistently evolves with age across individuals and even tissues (Tangili et al. 2023), although the underlying mechanisms are not yet well understood (Piferrer and Anastasiadi 2023). Certain differentially methylated sites across ages (referred to as “aging sites” hereafter) even share common characteristics among species (Klughammer et al. 2023), which has made it possible to fit universal epigenetic clocks for 185 mammal species (Lu et al. 2023; Robeck et al. 2021) and four fish species separated by up to 433 million years (Mayne et al. 2020, 2021). Methylated nucleotides were first detected through DNA modifications using antibodies, restriction enzymes, and bisulfite treatment (restricted to cytosine methylation; Anastasiadi and Piferrer 2023). The direct detection of methylated bases without altering DNA is now possible using third‐generation sequencing, as methyl groups produce distinct electrical patterns (Nanopore; Wescoe, Schreiber, and Akeson 2014) or speed patterns (PacBio; Flusberg et al. 2010). This approach also offers the potential to reliably discriminate among various methylation types: 4mC, 5mC, 5hmC, and 6 mA currently (Liu et al. 2021).

In parallel to the epigenetic and third‐generation sequencing breakthroughs in recent years, environmental DNA (eDNA) detection has revolutionized the assessment of biodiversity (Beng and Corlett 2020; Díaz‐Ferguson and Moyer 2014; Polanco et al. 2021). Detecting transient DNA traces of animals in water, sediments, or digestive systems is now an affordable and non‐destructive method that has been applied to swiftly and simply monitor communities' biodiversity (Beng and Corlett 2020; Rishan, Kline, and Rahman 2023). Notably, eDNA has various advantages for population genetic structure assessments, which are as crucial to demographic/dispersal models as age (Adams et al. 2019; Rishan, Kline, and Rahman 2023; Yao et al. 2022). While eDNA sampling is becoming more efficient and standardized, a major limitation is that it is not capable of obtaining individual‐scale information, such as sex, age, and condition (e.g., hormones and isotopes), or individual counts (Adams et al. 2019; Beng and Corlett 2020). eDNA epigenetics assessed from full‐length sequences present in the environment, using third‐generation sequencing instead of short metabarcoding regions, has the potential to overcome some of these challenges (Yao et al. 2022). Its ability to discriminate among the four life stages of the freshwater snail *Lymnaea stagnalis* has already been demonstrated (Zhao, van Bodegom, and Trimbos 2022).

Age is particularly important for stock assessments of bony fishes (Actinopterygii; Punt, Allen Akselrud, and Cronin‐Fine 2017), as over 50% of fish meat still comes from wild stocks (> 2200 species; Boyd, McNevin, and Davis 2022). This sets these fishes apart from most other heavily exploited taxa, emphasizing the need for proper management to prevent collapse in the face of the growing demand (Boyd, McNevin, and Davis 2022). Counting daily (in early life stages) or seasonal increments of otoliths (ear stones) is the standard age assessment method for fishes, and it has been estimated that several millions of otoliths are aged each year (Campana and Thorrold 2001). However, this method involves killing the fish, does not work for all fish species, suffers from various methodological biases, and is highly time‐consuming (Piferrer and Anastasiadi 2023). These drawbacks have prompted several attempts to improve this process through automated annotation and/or spectroscopy (Benson et al. 2023), as well as the use of radiocarbon/radiometry aging for a maximum resolution of about 1 year in adults (Piddocke et al. 2015). Despite the importance of developing new aging methods for fish species, only a few studies (Anastasiadi et al. 2018) on epigenetic clocks have been conducted with fish (e.g., Anastasiadi and Piferrer 2020), in contrast to 100 studies for wild mammals (Le Clercq et al. 2023). Considering wild fish species in particular, two studies showed that epigenetic clocks had uncertainty ranges comparable to those observed with otolith and radiocarbon aging, which are commonly used as calibration methods (Mayne et al. 2023; Weber et al. 2022). Epigenetic clocks might even be more accurate than other methods for early life stages, as they are characterized by a higher rate of methylation change (Bertucci et al. 2021). This is suggested by the higher accuracy of epigenetic clocks fitted to juveniles compared with those for adult fish (Anastasiadi and Piferrer 2020; Mayne et al. 2023).

In this study, we used eDNA to fit an epigenetic clock, taking advantage of two promising features of nanopore sequencing that have not yet been applied for the detection of animals through eDNA and aging: amplification‐free sequencing and detection of multiple methylation types at the single‐nucleotide scale. We focused on the larvae of seabass ( *Dicentrarchus labrax* ), as it was the first fish species for which an epigenetic clock was fitted based on tDNA (Anastasiadi and Piferrer 2020), and the environmental influence on methylation dynamics in early life stages is particularly well studied for this species (Anastasiadi, Díaz, and Piferrer 2017; Valdivieso, Sánchez‐Baizán, et al. 2023; Valdivieso, Anastasiadi, et al. 2023). Obtaining information about the age structure of wild seabass populations is of major conservation importance because stocks have greatly declined in recent years (de Pontual et al. 2023), and it is a species of major economic interest (sixth most farmed marine bony fish in the world; 244 kT fished in 2020; FAO 2022). Studying fish larvae is of crucial importance because it is the most vulnerable stage, and survival during this stage determines population replenishment while ensuring population connectivity for most demersal fish species (Fontoura et al. 2022). Culturing *D. labrax* allowed us to study larvae within a controlled environment, to reduce the number of technical challenges for this preliminary study (Yao et al. 2022). Apart from the overall aim of determining whether accurate epigenetic clocks can be fitted from eDNA, we pursued three research questions: (1) Are methylation‐based age assessments effective for early life stages? (2) What is the potential of nanopore sequencing for detecting methylated patterns, and (3) Are there statistical methods available that would increase the efficiency of epigenetic clock fitting?

## 2 Materials and Methods

### 2.1 eDNA Sampling

**FIGURE 1:**
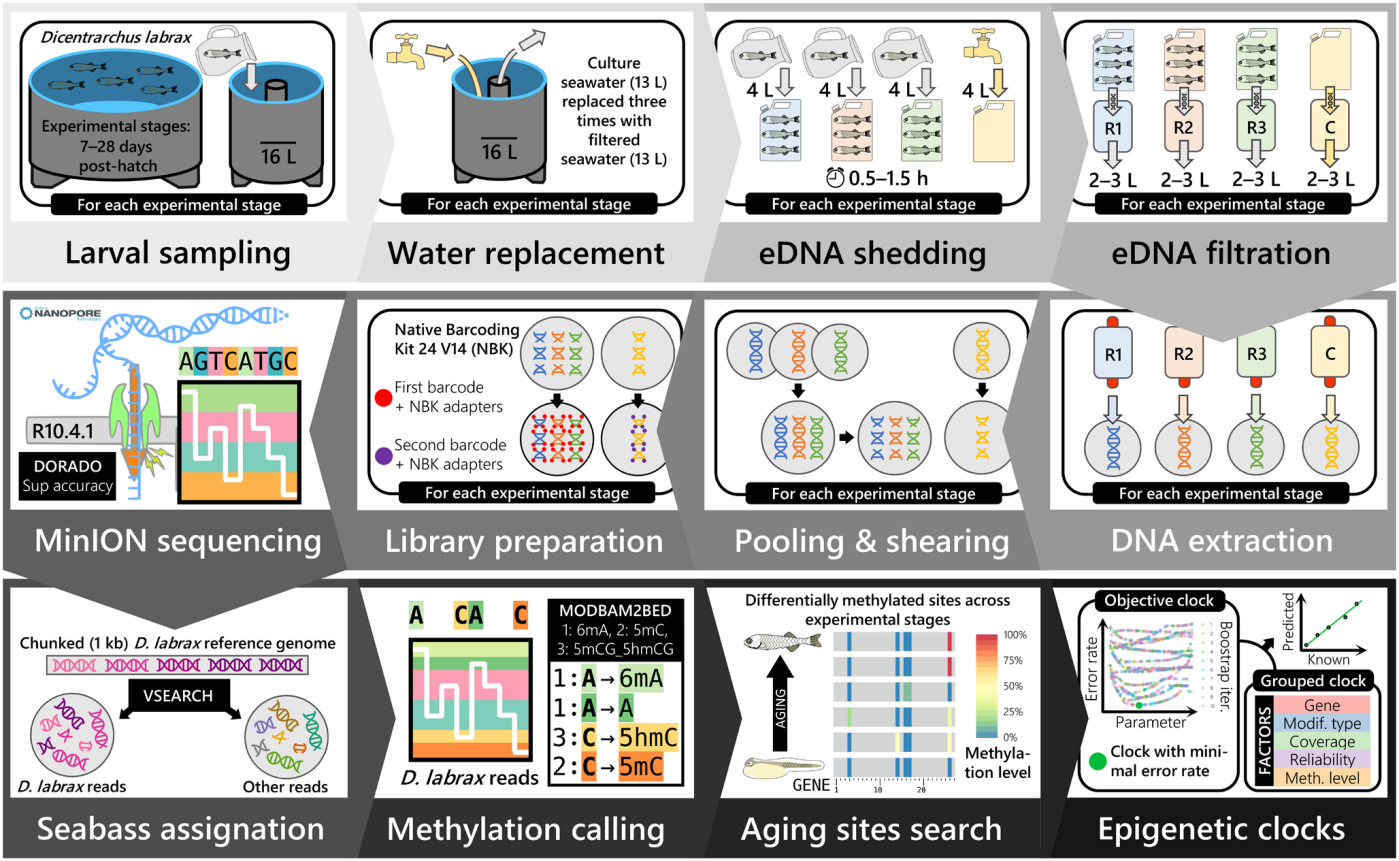
Experimental workflow, from environmental DNA (eDNA) sampling in the aquaculture facility (top row) to eDNA sequencing in the laboratory (middle row) and subsequent bioinformatic analyses for epigenetic clock fitting (bottom row). *Dicentrarchus labrax* (seabass) larvae were kept in filtered seawater for 0.5–1.5 h to maximize the proportion of seabass eDNA retrieved in 0.45 μm Sylphium filters for each experimental stage (i.e., 7, 10, 12, 14, 17, 19, 24, 26, and 28 days post‐hatch). Due to the small amount of eDNA extracted for most samples, replicates (R) were pooled together, and further sheared to increase sequencing yield using the latest multiplexing chemistries (NBK‐24V14), flow cells (R10.4.1), and basecalling algorithms (DORADO: Dna_r10.4.1_e8.2_400bps_sup@v4.2.0) currently available from Oxford Nanopore Technologies. Methylation calling for seabass reads only was performed using three submodels of the Dorado basecalling algorithm, and aging sites were further detected from MODBAM2BED summaries for each modification type. Four variables describing aging sites and their respective methylation level across ages were used to fit epigenetic clocks using a new method coupling grouped penalized regularizations with objective optimal model selection criteria (minimal error rate) across model parameter values and bootstrap iterations.

We carried out our experiments on a pool of seabass larvae ( *Dicentrarchus labrax* ) cultured in the aquaculture facility of the OREME (Mediterranean Coastal Environment Station) observatory (Sète, France). After an initial growth phase of a pool of larvae born on the same day in a nearby fish hatchery (Fermes Marines du Soleil, Balaruc‐les‐Bains), larvae were delivered to the OREME at 5 days post‐hatch (DPH). Subsequently, their density in the aquaculture tank was assessed daily. We collected eDNA from 7 DPH (18/11/2022) to 28 DPH (09/12/2022) every 2 or 3 days for a total of 10 sampling days (Figure 1). However, due to a technical issue, the sampling could not be conducted at 21 DPH (02/12/2022), resulting in a total of nine different ages (7–28 DPH).

To limit human contamination of the samples, we conducted each experiment in a dedicated aquaculture zone, where it was mandatory to wear latex gloves, surgical masks, laboratory coats, and hair nets. The first phase of the experimental procedure involved decontaminating all sampling materials and tanks with bleach (10%), then rinsing them with distilled water. We then used a 2 L beaker to transfer 16 L of seawater containing seabass larvae from the aquaculture tank to a bleached tank with a tap fitted near its base, which was topped with a suction strainer. We released about 13 L of seawater through the tap before refilling the tank with filtered seawater, which had been pumped at sea and filtered through a 1 μM mechanical filter. We repeated this operation three times to ensure that most seawater from the aquaculture tank had been eliminated. Then, using a 1 L beaker, we transferred 4 L of filtered seawater containing larvae into three 5 L canisters (replicates), whose screwed caps were sealed using parafilm. As a control measure to monitor potential DNA contamination of the samples, we filled another canister closed similarly and filled only with 1 μM filtered seawater from the aquaculture station. We left it in the same conditions than the replicates (i.e., positioned next to them; Figure 1).

After 30 min, we filtered a volume of 2–3 L from each canister through a Sylphium eDNA 0.45 μM dual filter capsule using an Athena peristaltic pump (Proactive Environmental Products LLC, Bradenton, Florida, USA; nominal flow of 1.1 L min^−1^) and disposable sterile tubing for each filter capsule. The filtered volume was only 1.3 L for one replicate at 7 DPH, as the filter clogged too quickly, and it was increased from 2 to 3 L after 12 DPH, due to lower larval densities. The filtering was performed sequentially by a single person, which means that larvae had 0.5–1.5 h to shed eDNA (duration noted for each filter). At the end of each filtration, we emptied the water inside the filter capsules and then filled the capsules with 5 mL of Longmire lysis buffer solution.

### 2.2 eDNA Extraction

We extracted DNA from all filter capsules within 2 weeks after the end of the experiment. One replicate at 7, 10, and 26 DPH could not be processed because the filter tip was broken during transport and all liquid leaked out. We carried out the eDNA extractions in facilities dedicated to this purpose, following a protocol adapted from Pont et al. (2018). Modifications were as follows: We retrieved 2 mL of buffer for the extraction; when removing the supernatant, we left 0.6 mL of liquid at the bottom and then mixed it with 1.32 mL of absolute ethanol and 60 μL of 3 M sodium acetate; and we performed the final elution step with 2 × 50 μL SE buffer. We measured DNA concentrations with the Qubit high sensitivity dsDNA kit (Thermo Fisher Scientific, Bremen, Germany) following the manufacturer's instructions. We detected no contamination during extraction, as blanks for each round of extraction presented a DNA concentration below the Qubit detection limit (< 0.0005 ng μL^−1^). Finally, we stored the filter extracts at −20°C until further processing.

### 2.3 eDNA Preparation and Sequencing

We pooled the extracts from the three sample replicate filters in 1.5 mL Eppendorf tubes to increase the amount of total DNA for sequencing. In cases where one sample was unavailable due to leaked filters, only two were pooled, resulting in a total of nine pooled samples (P1–P9) and nine negative controls (C1–C9) ordered by age (7–28 DPH). Following the pooling procedure, the total DNA in most pooled samples remained below the required 400 ng per sample for Oxford Nanopore Technologies (ONT) sequencing (Table S1 in Data S1). However, three pooled samples met the sequencing criteria (Table S2 in Data S1).

Following advice provided by Oxford Nanopore Technologies in the case of low DNA input, we fragmented the DNA molecules to ensure that a sufficient number of molecules would pass through the nanopores and that none would be blocked. We concentrated each sample to 50 μL by heated evaporation (2 h, 30°C) using a Concentrator 5301 (Vaudaux‐Eppendorf, Schönenbuch, Switzerland). We added 15 zirconium oxide beads (1.4 mm; Precellys P000927‐LYSK0‐A, Bertin‐Instruments, Montigny‐le‐Bretonneux, France) to each tube, placed the tubes in a MM 301 homogenizer (Retsch, Haan, Germany), and agitated them twice for 10 s at 30 Hz, inverting tube positions between the two runs to ensure symmetrical treatment of all samples. We recovered as much supernatant as possible (typically ~45 μL, i.e., 90%) and centrifuged it for 1 min at 21,300 × *g* to pellet proteins. Again, we kept only the supernatant for the next steps, discarding the protein pellets. DNA contents measured using the Qubit high sensitivity dsDNA kit (Thermo Fisher Scientific) revealed that only two pooled samples contained the amount of total DNA required for ONT library preparation (Table S2 in Data S1).

We then concentrated the samples to 11 μL, using the same heated evaporation method as above (45 min, 30°C). Subsequently, we prepared the library using the Native Barcoding Kit 24V14 (SQK‐NBD114.24, Oxford Nanopore Technologies, Oxford, United Kingdom) according to the ligation sequencing gDNA native barcoding v14 protocol from ONT, using a short fragment buffer during adapter ligation and cleanup and adding BSA (Bovine Serum Albumine) during flow cell flushing. P8 and P9 were inadvertently mixed during library preparation, prohibiting differentiation between the last two experimental stages (26–28 DPH). We analyzed the final library on an TapeStation 4150 (Agilent, Santa Clara, United States) using Genomic DNA ScreenTape, which showed a peak fragment length of 7 kb (94.19% of fragments were between 3058 and 23,454 bp). We loaded and ran the library on an R10.4.1 flow cell (FLO‐MIN114, ONT) on a MinION Mk1B (MIN‐101B, ONT) sequencer (Figure 1).

### 2.4 Basecalling and Methylation Calling

We basecalled the sequencing files not filtered by their quality score using the latest and most accurate basecalling model of ONT (dna_r10.4.1_e8.2_400bps_sup@v4.2.0) available to date in the open‐source basecaller for Oxford Nanopore reads, Dorado. We demultiplexed the file generated using the guppy_barcoder program and subsequently aggregated all generated outputs per barcode and converted the aggregate to a single FASTA file, keeping track of read names and barcodes.

We detected seabass reads among all the other sequenced reads using a 90% threshold of minimum similarity in VSEARCH (Rognes et al. 2016) to a reference seabass whole genome. We downloaded this genome from NCBI (RefSeq: GCF_905237075.1, Tine et al. 2014), which was chunked in 1 kb fragments to speed up the assignation on a CPU server and obtain the most assigned reads (Figure S14 in Data S7). We selected this similarity threshold because it corresponds to the optimal gap to delineate genera for many mitochondrial genes (Ruiz et al., *in prep*), enabling the identification of all *Dicentrarchus* reads while accounting for potential basecalling errors. We verified the absence of the only other species in this genus ( *D. punctatus* ) using various assignation methods (Data S2 and S3). Additionally, we replicated analyses using larger (10 kb) reference fragments (i.e., 9000 shared nucleotides instead of 900). This resulted in the identification of about two times fewer aging sites, due to the smaller number of seabass reads detected (Figure S15 in Data S7).

After these cleaning steps, we performed the methylation calling on seabass reads using the submodels 5mC, 5mCG_5hmCG (CG meaning in the CpG context only), and 6 mA integrated in the Dorado program. We mapped the output of the methylation calling both on the reference mitogenome (RefSeq: NC_026074) and on the full reference genome (containing this mitogenome) using the aligner program from Dorado, as well as the sort and index programs from SAMtools (Danecek et al. 2021). We then used the modbam2bed program from ONT to generate methylation summaries based on the mappings across the whole reference genome for each type of modification (i.e., modC, 5mC, 5hmC, modA, and 6 mA) detected separately by each submodel (eight combinations).

From these analyses, we obtained the methylation summary for each mapped site (e.g., modification type, reliability score, coverage, and methylation level), and we summarized this information for each barcode using R version 4.1.1 (Data S5). As samples collected at 7 DPH (i.e., too few reads in barcode 1) and 26/28 DPH (i.e., mixed Barcodes 8 and 9) were not suitable for further analyses, we used R to identify aging sites as differentially methylated sites (i.e., at least one unequal methylation level) across the Barcodes 2–7 (10–24 DPH).

### 2.5 Mapping Analysis

For each type of mapping (i.e., mitogenome or full genome), we summarized the correspondence between the reference genome and seabass reads, potentially reflecting natural intraspecific variations or basecalling errors, using the “read accuracy” and “read identity” concepts introduced with third‐generation sequencing. We applied a modified version (i.e., no implementation of read identity and a different formula for read accuracy) of an R function (import_bam_file) provided by Gleeson et al. (2022) to convert the file from the methylation calling into the concise alignment format CIGAR, from which we computed both metrics. First, we calculated the read accuracy, which corresponds to the ratio of the number of matches to the alignment length. Second, we computed the read identity as the ratio of the number of matches to the number of bases aligned (i.e., not considering gaps). This definition was the most useful in our case because we did not expect any nucleotide insertion or deletion caused by basecalling errors (Data S4).

### 2.6 Epigenetic Clocks

We fitted epigenetic clocks using penalized regularizations (lasso, ridge, or elastic‐net regressions) that individually shrank the weight of each aging site since their number was much greater than the number of ages in this study. To determine if factors other than methylation levels had to be taken into account when using grouped penalized regularizations, we preliminarily used a permutational multivariate analysis of variance (PERMANOVA) and a Mantel test to check for any significant links between methylation levels and variables characterizing aging sites (Data S6). Based on these tests, we decided to fit epigenetic clocks taking into account all four variables characterizing the tested aging sites: (1) the gene obtained using a custom R function from the GenBank file of the reference mitogenome (NC indicating “non‐coding” for non‐annotated sections), (2) the modification type (5mC, 5hmC, Other modC, and 6 mA), (3) the mean coverage across experimental stages, and (4) the reliability score, taking into account the chances of confusion with another modification type for each read, which would ultimately affect the modification frequency.

First, we objectively chose the best values of the penalty factor α by testing each value between 0 and 1 by increments of 0.01 to fit both ridge regression (*α* = 0) and lasso regression (*α* = 1), as well as elastic‐net regression for all values of α in between 0 and 1. Second, we objectively determined the optimal number of folds for the cross‐validation, although in our case with only six different ages we could only use a number of three (also not split into training/testing datasets). Third, due to large variability between models fitted on the same values, and to ensure convergence toward a stable optimal model (Figure S4 in Data S3), we employed bootstrapping (10 parallelized iterations) to fit epigenetic clocks for all combinations of penalty factors and numbers of folds. Fourth, along with modification frequency, we considered all possible combinations of the four factors mentioned above (Figure 5), by pasting the considered features so that sites with the same label were grouped together for regularization. Finally, we selected the best models, defined as those with the lowest median absolute error (MAE) during the cross‐validation, as well as the lowest MAE during the final prediction, using the full training dataset as input. We standardized these two metrics by giving them the same weight and then summed them to select the best model, that is, the one with the lowest overall score (Data S6). We created two R functions based on the “glmnet” (Friedman, Hastie, and Tibshirani 2010) and “grpnet” (Helwig 2023) R packages to change fitting/selection criteria and make profit of a trained model to predict new ages from a testing matrix (see commented functions on Github: https://github.com/ruizeliot/eDNA_epigenetic_aging_seabass_2024).

We fitted epigenetic clocks both on the full dataset and on datasets containing only methylated adenosines (6 mA) or only methylated cytosines, which could be 5mC, 5hmC, or modC that were not characterized as 5mC or 5hmC by both models (named “Other modC”). We removed sites characterized as 5mC by the 5mC submodel and as 5hmC by the 5mCG_5hmCG submodel (six in total).

## 3 Results

### 3.1 Nanopore Sequencing of Seabass Larvae eDNA

The amount of eDNA extracted averaged 303 ng per sample, but the mean coefficient of variation between experimental stages (87%), and even between replicates (69%) was very high. A robust linear regression revealed that the estimated biomass of larvae per canister was the best predictor of the extraction yield, compared with the duration of eDNA shedding, the volume filtered, or the larval age (*t*(19) = −4.754, *p* < 0.001, *r*_s_ = 0.28; Figure S1 in Data S1). The DNA input was lower than the recommended 400 ng for ONT library preparation, both for control samples (*μ* = 99, *σ* = 89 ng) and for most pooled testing triplicates (*μ* = 260, *σ* = 333 ng), notably due to a great loss during shearing (*μ* = 77%). Nevertheless, we were able to obtain a total of 1.8 million reads, with an average length of 1.35 (*σ* = 1.85 kb). Neither the total number of reads (*t*(16) = 0.230, *p* = 0.82) nor the number of reads (*t*(16) = −0.192, *p* = 0.85) presenting a similarity ≥ 90% to the 1 kb chunked reference seabass genome (“seabass reads”) were significantly linked to the DNA input weight (Figure S2 and Table S3 in Data S1).

Seabass reads were found in all control and test samples (Table S3 in Data S1), but their mean proportion of the total number of reads per sample was much higher for test samples (*μ* = 6, *σ* = 5%) than for control samples (*μ* = 1, *σ* = 2%). Complementary blasts of all fast basecalled seabass reads (*Q*‐scores > 8) to all *D. labrax* reads in the NCBI nucleotide database confirmed that there was slight contamination with seabass nuclear DNA of at least five control samples (Data S2). However, after comparing assignations with similarities ≥ 90% of superior basecalled reads in control samples to the reference full genome and mitogenome (Data S3), we chose to neglect this contamination with eDNA from seabasses of unknown age, since 99.9% of control contamination occurred in discarded experimental stages and for nuclear DNA.

The two approaches using the whole NCBI nucleotide database or reference complete mitogenomes (i.e., RefSeq database from NCBI) as targets during the assignation of fast and superior basecalled reads, respectively, were also adopted to identify non‐seabass reads. Human DNA contamination was successfully minimized during sampling (e.g., 0.1% of total eukaryotes' mitogenomic reads). However, our protocol—carried out in a non‐sterile environment using 1 μM filtered seawater—was hampered by airborne and waterborne contamination from microorganisms (i.e., > 30% of bacteria and > 23% of fungi), as 94% of the reads obtained in test samples with amplification‐free nanopore sequencing were not assigned to *D. labrax* (Data S2 and S3).

**FIGURE 2:**
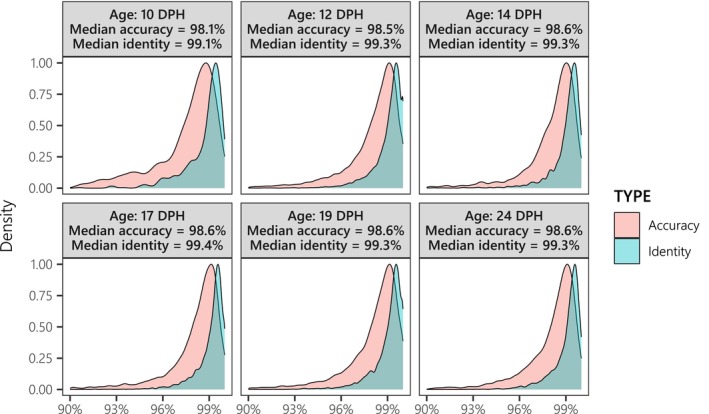
Density functions of the read accuracy (red) and the read identity (blue) per sample, computed from mappings of *Dicentrarchus labrax* (seabass) reads identified with VSEARCH on the reference *D. labrax* whole genome. Unlike read accuracy, read identity does not take into account alignment gaps, which makes it a more suitable performance metric for this study because basecalling errors should not cause additional insertions and deletions (see Data S2).

Although a correct quantification of the read accuracy was not possible since we did not know the genomes of our specific seabass population, the read identity showed that there was at least < 0.7% (median) of basecalling errors both for all fragments (Figure 2) and for mitogenomic fragments only (Figure S6 in Data S4). Longer reads had a significantly lower read identity, and the other significantly correlated variables in a robust regression were those characterizing the alignment quality with the reference genome (Figure S7 in Data S4).

### 3.2 Detected Aging Site Types

Taking into account the reliability score, we detected a methyl group on 0.8% of the mapped cytosines or adenosines, which yielded a mean per test sample of interest (10–24 DPH) of 99,187 modA sites and 154,772 modC sites (Figure S8 in Data S5). By comparing outputs from the 5mC and 5mCG_5hmCG submodels, we estimated that about 84% of modC sites were in a CpG context, and 5.6% of these CpG sites were hydroxymethylated (5hmC could only be detected in a CpG context at the time of analysis). Another fraction (not quantified) of modC sites in CpG contexts only (5mCG_5hmCG) was categorized as neither 5mC nor 5hmC.

**FIGURE 3:**
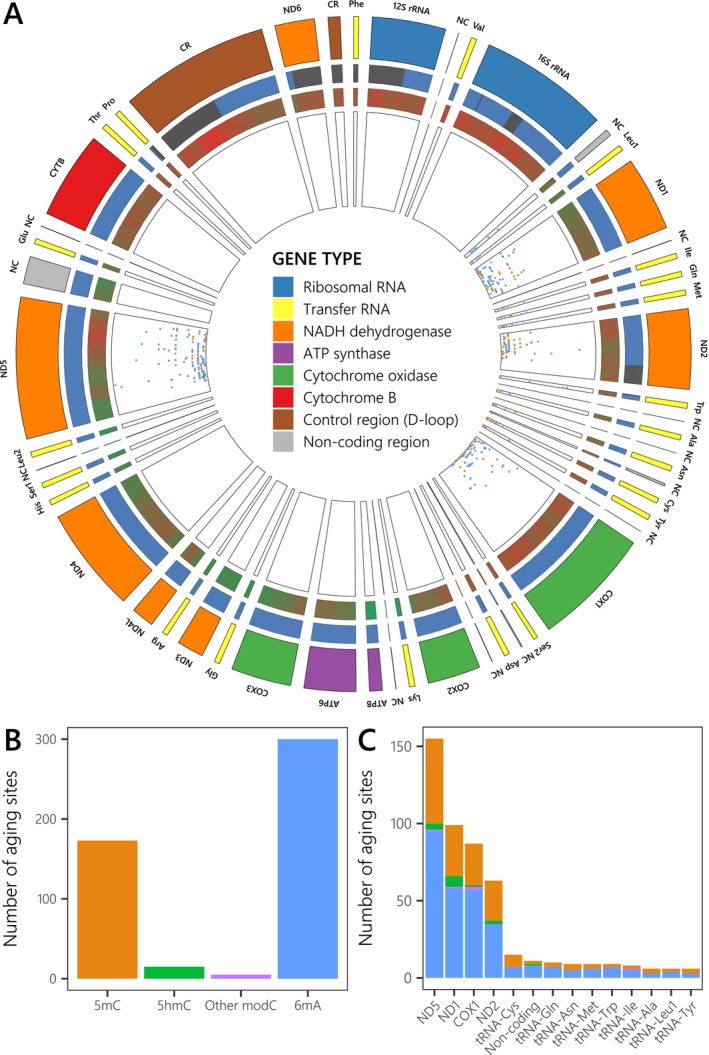
(A) Coverage and position of aging sites on the reference *Dicentrarchus labrax* mitogenome. The position of a dot on the inner ring corresponds to the mean methylation level across the samples from 10 to 24 days post‐hatch (DPH) used to fit epigenetic clocks, from 0% (closest to the center) to 50% (farthest from the center). (B and C) Sum of aging sites detected per modification type (B) and per mitochondrial region (C). The colors of the dots (A) and stacked bars (B and C) correspond to the methylation types: 5mC (orange), 5hmC (green), other modC (purple), and 6 mA (blue). Moving outwards in (A), the next ring represents the summed coverage per mapped position, from 11× (dark red) to 27× (dark green). In the following ring, sites covered for all ages of interest (10–24 DPH) for which it was possible to search for differentially methylated sites across ages (i.e., aging sites) are represented in blue, while their counterparts are shown in gray. The outer ring corresponds to the different genes and non‐coding regions (NC) of the reference *D. labrax* mitogenome, colored by gene type as indicated in the legend. This figure can be viewed interactively on: https://eliotruiz.shinyapps.io/eDNA_methylation_RUIZ_ET_AL_2023/.

**FIGURE 4:**
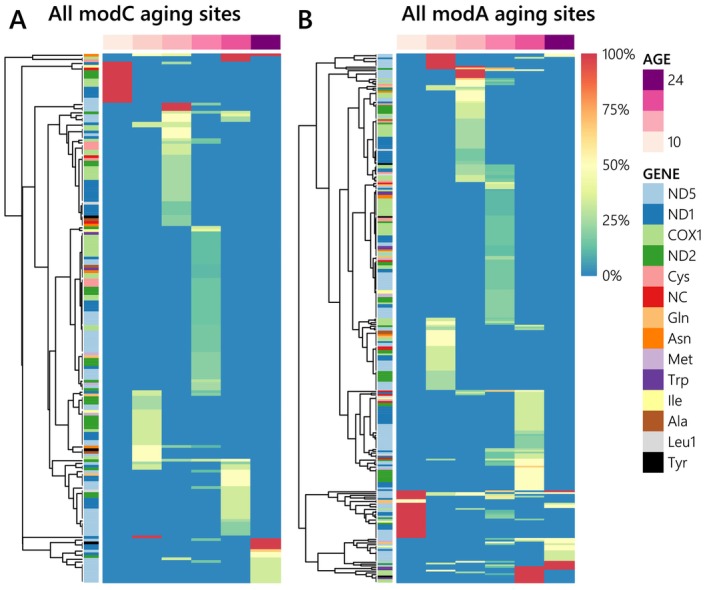
Heatmaps of the methylation level per sample (columns) of all modified cytosine (A: 5mC, 5hmC, and other modC) and all modified adenosine (B: 6 mA) aging sites detected (rows). Aging sites presenting a similar methylation pattern across ages were clustered together (dendrogram), and the genes on which they are located are represented by different colors next to the dendrogram.

Unlike methylated sites, there were more modA than modC aging sites (ratio from 0.64 to 1.55) for a total of 493 aging sites, and the proportion of 5mC over all modC sites also decreased from 97.7% to 89.6% compare to all methylated sites (Figures 3B and S8 in Data S5). We only detected aging sites on the mitogenome which represents less than 0.06% of the mapped cytosines and adenosines (Figure 3A). Indeed, mitogenomic mapped sites and aging sites had a mean coverage of 3.00× instead of 1.01× if all mapped sites were considered, which had a large significant effect in a non‐parametric analysis of variance (ANOVA; *F*(1,36) = 44.55, *p* < 0.001, $\;\eta_{p}^{2}=0.60$) compared with the modification type and the type of sites (i.e., candidate or aging sites), which were not significant (Data S5). Aging sites also seemed to have a non‐random distribution in the mitogenome because they were found only on certain genes, and even certain gene portions, despite having a similar coverage across ages for most other genes (Figure 3A). They mainly occurred on codon‐organized genes (i.e., 73% of aging sites; Figure 3C), such as NADH dehydrogenase subunits 1 and 5 (ND1 and ND5) genes and the cytochrome c oxidase subunit I (COX1) gene (ND2 to a lesser extent). Nevertheless, the average density of aging sites per gene (7.5% vs. 14.7% of gene length) was two times higher on the nine much smaller tRNA genes and even on two supposedly non‐coding sections (Figures S10 and S11 in Data S5). Aging sites were generally methylated for a single experimental stage (no sites were methylated throughout the entire experiment), and most methylation levels of 100% occurred at 10 DPH, without clear methylation patterns per gene or modification type (Figure 4). However, the methylation level was significantly linked to both qualitative (gene and modification type) and quantitative factors (coverage and reliability score; Figure S12 in Data S6), either per experimental stage (Mantel Test, *p* < 0.001 for all variables) or averaged across the experiment (PERMANOVA, *p* < 0.05 for all single variables except the type only significant in interaction with genes: *p* = 0.02), even though standardized effect sizes ($\omega_{p^{2}}=0.11$) were categorized as small for all variables except the gene (Data S6).

### 3.3 Efficient Prediction of Larvae Ages

**FIGURE 5:**
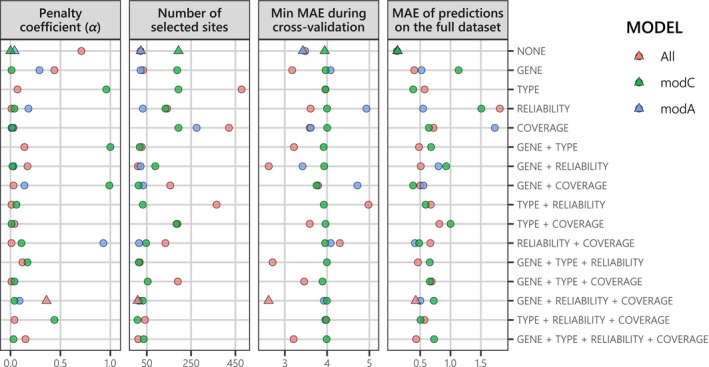
Summary metrics for each optimal epigenetic clock per grouping combination (rows: From no grouping to all position features pasted together as groups) and per training dataset (colors: All aging sites, or only modified cytosine [modC] or modified adenosine [modA] aging sites). Each dot corresponds to the epigenetic clock with the lowest minimum median absolute error (MAE) during cross‐validation and during the final prediction of known ages using the full dataset among all that were fitted per *α* increment from 0 to 1 (conditioning the number of sites with a coefficient different from 0, that is, selected sites) and per bootstrap iteration from 1 to 10. Among all optimal epigenetic clocks (dots), the best one (triangle) for each type of training dataset was selected using the same double MAE criterion as described above.

To take into account all these factors, along with methylation levels, for seabass age prediction, we developed a method to fit an objective epigenetic clock based on grouped penalized regularizations (i.e., lasso, ridge, or elastic‐net regressions) systematically tested for minimum error rate across penalty factors (α) and bootstrap iterations. Indeed, we detected complex relationships between α values and the resulting model performance, which greatly varied between bootstrap iterations (Supplement 6). Using 10 bootstrap iterations, we were able to reach stable optimal performances of epigenetic clocks fitted across all possible combinations of additional factors and across the three datasets (Figure 5). Overall, most optimal *α* values were close to 0, yielding a large number of non‐neutralized “selected” sites, even though the link between α and the number of selected sites was not straightforward because the sites' coefficients were set to be very close to, but not at, 0 (Figure 5). The minimum MAE during cross‐validation was very similar (around 4 days) among groupings for clocks fitted on the modC dataset, while it varied greatly (between 2.6 and 5 days) for the two other datasets (Figure 5).

**FIGURE 6:**
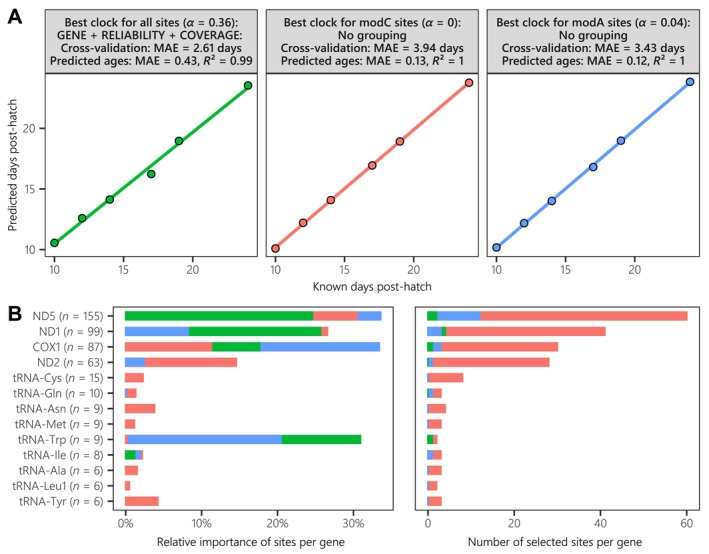
(A) Linear regression of predicted versus known ages per sample using the best epigenetic clock (triangles in Figure 6) per training dataset, shown with the corresponding parameters and summary metrics. (B) Proportion of summed absolute coefficients of all aging sites (*n*) per gene out of the total, per best epigenetic clock (i.e., relative importance of genes), as well as the number of sites with a coefficient different from 0 (i.e., selected sites) per gene.

MAE was much lower after training when no groups were used (around 0.1 days), while this metric was between 0.5 and 1 day for most other clocks. The best epigenetic clocks selected for the modC and modA datasets were therefore fitted without groupings (Figure 5). Conversely, epigenetic clocks fitted on the full dataset that accounted for the gene, the mean reliability score, and to a lesser extent the mean coverage had the best results in terms of cross‐validated MAE (Figure 5). Indeed, we obtained a cross‐validated MAE reaching 2.61 days in this case, compared with 3.94 and 3.43 days for the smallest modC and modA datasets, respectively. Despite such a result, the MAE after training was slightly lower for the full dataset (0.43 days), even if the overall correspondence between known and predicted values was high (*R* ^2^ = 0.99, Pearson correlation coefficient = 1; Figure 6A). To characterize the importance of each gene in the age prediction, we summed the absolute coefficients of their respective sites instead of just accounting for the number of selected sites per gene, which was more linked to the penalty factor chosen (Figure 6B). Generally, the most important genes for each clock were those with the largest number of aging sites with high methylation levels per dataset (especially for the modC best clock with *α* = 0), even though the tRNA‐Trp gene had a very high importance—despite having only nine sites—compared with codon‐organized genes (especially the ND2 gene; Figure 6B). Indeed, seven aging sites on the tRNA‐Trp gene were 6 mA, and most had a methylation level of 0% at 10–14 DPH, followed by an average increase of 8.5% at 17 DPH, before reaching 100% at 19 DPH and 24 DPH for three and two sites, respectively (Figure 4B). Overall, mitogenome methylation seems sufficient for the accurate age prediction of the seabass larvae studied here.

## 4 Discussion

Our study shows that eDNA can be used to fit epigenetic clocks, but also that their accuracy might be equivalent to, or even better than, most other clocks previously established. We obtained the highest correlation (Figure 6) of all methylation‐based (*μ* = 0.92, 95% CI [0.89; 0.94]) and telomere‐based (*μ* = 0.31, 95% CI [0.25; 0.37]) clocks listed in the review by Le Clercq et al. (2023). The cross‐validated median absolute error (MAE) obtained in our analysis with the best clock (2.61 days) considering all aging sites exceeded previously obtained cross‐validated MAE values for non‐model animals (*μ* = 1.28 years; Tangili et al. 2023). However, the MAE scaled to the age range was twice as high (i.e., 0.19 vs. *μ* = 0.08) due to the very short age range considered here. The improvement in cross‐validated MAE between the clock previously established for *D. labrax* with DNA tissue using a classic workflow (140 days; Anastasiadi and Piferrer 2020) and our clock based on nanopore sequencing of eDNA highlights their respective potentials in the field of eDNA epigenetics. The detection of aging sites only on the mitochondria suggests that eDNA release might be an alternative demethylation mechanism (Zhao, van Bodegom, and Trimbos 2022). Our study paves the way for further developments of eDNA epigenetic methodologies, which is promising for conservation and management applications (Yao et al. 2022).

The higher accuracy of this new epigenetic clock for larval *D. labrax* compared with those for juveniles and adults of the same species, both in term of MAE (2.6 days vs. 2.1 years) and scaled MAE (0.19 vs. 0.21; Anastasiadi and Piferrer 2020), supports the hypothesis that early life stages allow more accurate epigenetic clocks to be fitted than older stages. A substantial reduction in cross‐validated MAE between juvenile and adult clocks has already been observed in studies about fishes involving various life stages of *D. labrax* (Δ = 182 days; Anastasiadi and Piferrer 2020) and *Macquaria ambigua* (Δ = 644 days; Mayne et al. 2023), the latter being the most accurate clock to date other than ours (MAE = 3.5 days; scaled MAE = 0.004). The great potential for aging young fishes with dedicated clocks is corroborated by various studies that detected a faster rate of methylation change (Bertucci et al. 2021) and a greater dependence on the environment for early life stages (Suarez‐Bregua et al. 2020; Wu et al. 2018), which is particularly important for seabass larvae (Anastasiadi, Díaz, and Piferrer 2017; Valdivieso, Anastasiadi, et al. 2023). The very rapid change in methylation levels per site which apparently does not follow the gradual trend toward hypermethylation with aging generally observed in fishes (Bertucci et al. 2021) and other animals, corresponds, however, to patterns observed in all four studies on methylation changes during the larval phase of fishes (Wu et al. 2018; Suarez‐Bregua et al. 2020; Suarez‐Bregua et al. 2021), including *D. labrax* (Anastasiadi, Díaz, and Piferrer 2017). Under the rearing conditions we used to grow seabass larvae, developmental changes between 10 and 24 DPH are indeed numerous, involving notably exogeneous feeding and fin development starting at 8 DPH, as well as glass bladder apparition and branchial respiration from 10 DPH. In general, the larval phase seems to constitute a plastic period during which larvae from the same population sharing a very similar genome might encounter very different environmental conditions, and therefore adapt their gene expression to the local conditions. This is illustrated by regulations of sex (Anastasiadi et al. 2018; Valdivieso, Anastasiadi, et al. 2023) and growth rates depending on the rearing temperature (Anastasiadi, Díaz, and Piferrer 2017; Burgerhout et al. 2017; Campos et al. 2013) in various species, including *D. labrax* . Both ecological and morphological metamorphosis, which might be decoupled in some demersal fish families (Richards and Lindeman 1987), have also been shown to be associated with methylation changes for metamorphic (Anastasiadi, Díaz, and Piferrer 2017; Suarez‐Bregua et al. 2020; Wu et al. 2018) and physiological remodeling (e.g., osmoregulation; Blondeau‐Bidet et al. 2023; Covelo‐Soto, Saura, and Morán 2015; Liu et al. 2022; Trautner et al. 2017). These epigenetic regulations not only enable the differentiation of life stages without the use of an epigenetic clock (Trautner et al. 2017), but also facilitate predictions of sex with great accuracy (~90%) if it is environmentally mediated (Anastasiadi et al. 2018; Valdivieso, Anastasiadi, et al. 2023). Environmental DNA epigenetics appears to be a promising tool to gather information for management purposes on key biological parameters for individuals present near the sampling area, instead of relying on expensive yearly ichthyoplankton surveys (e.g., van der Lingen and Huggett 2003).

Compared with previous approaches employed to detect methylated nucleotides, which involved microarrays or bisulfite sequencing in epigenetic clock studies (Tangili et al. 2023), nanopore sequencing has various advantages that might explain the low error rate of our clock. First, with this method it is possible to search for aging sites in a non‐targeted way, as it does not involve amplifying/enriching a specific region of DNA, and the method performs better at detecting CpG sites than the equivalent bisulfite‐based technique (i.e., WGBS; Liu et al. 2023). Second, it does not require any DNA modification, which can generate errors, and it enables the retrieval of long reads that are easier to map to a reference genome (Laine et al. 2023; Schatz 2017). Third, it allows detection of cytosine methylation in all contexts, while simultaneously detecting 5hmC and 6 mA modifications, unlike previous methods. In our case, this increased the number of methylated nucleotides (all: 254 K; CpG only = 123 K) and the number of aging sites detected by more than half (Figures 3B and S8D in Data S5). These two less‐studied modification types might even be informative since they are both thought to be involved in the aging process (Shi et al. 2017; Xie et al. 2023). They made up a larger proportion of aging sites versus 5mC sites compared with the equivalent proportion for methylated sites (35% vs. 52%; Figure 3B), and the adenosine‐based clock had a lower MAE than the cytosine‐based clock (3.43 vs. 3.94 days; Figure 6). The main criticisms of nanopore sequencing focus on its sequencing accuracy, but this has improved over the past few years, and we achieved < 0.7% in our study using the latest materials available (R10.4.1 + V14).

The high accuracy of our clock is due to our newly developed methodology, which fits optimal epigenetic clocks based on objective selection criteria, taking advantage of various qualitative and quantitative variables characterizing aging sites obtained with nanopore sequencing (Figure 1). Most epigenetic clocks utilize penalized regression to prevent overfitting, as the number of aging sites is often greater than the number of samples (Anastasiadi and Piferrer 2023), which requires manually tuning (e.g., *α* = 0.5; in fishes: Bertucci et al. 2021; Mayne et al. 2020) the parameter determining the amount of shrinkage (*α*). The use of a systematic and bootstrapped approach to objectively determines parameters reduced the stochasticity of performances for equal model parameters (Figure S13 in Data S6), but our results question the repeatability of performances between previous studies that have not yet been tested (Piferrer and Anastasiadi 2023). In our study, grouping aging sites that shared similar features before fitting an epigenetic clock made it possible to reduce error rates through the objective choice of the best groupings in the global model (i.e., gene and uncertainty‐associated variables; Figure 5). However, this approach is not necessarily the best for smaller subsets sharing similar modification types (Figure 6). Aside from nanopore sequencing, this approach could be generalized to account for any qualitative (e.g., tissue and CpG context) or quantitative (e.g., replicates and alignment/mapping scores) features, depending on the methylation assessment methodology, and could be applied to further explore if neighboring methylated sites influence each other in terms of characteristics (Laine et al. 2023). The main limitation of our method is that it only predicts age from evolving methylated patterns as an animal ages, without any insight into the underlying biological processes; informed deep neural networks could be a solution to this problem (Prosz et al. 2024). Another limitation specific to this exploratory study is the absence of an independent validation dataset, which forced us to predict new values from the same training dataset (Figure 6A). This likely led to a significant underestimation of the model's true performance (i.e., *R* ^2^ and final MAE). As solely using cross‐validation is common in machine learning studies and considered as safe (King, Orhobor, and Taylor 2021; Levman et al. 2023), we advocate, in line with Tangili et al. (2023), that only cross‐validated MAE obtained after testing on ⅓ of the initial dataset the model trained on the other ⅔ should be considered as reliable.

The exclusive presence of aging sites on mitochondrial DNA (mtDNA) is in contrast to the many methylated sites detected on nuclear DNA (nuDNA), and mtDNA generally makes up 0.1%–1% of extracted tDNA (Ramón‐Laca, Gallego, and Nichols 2023). This pattern might be explained by the observed coverage bias (Figure S9 in Data S5), in that it is more difficult to obtain sufficient coverage (nuDNA: 1×; mtDNA: 3×) to detect differentially methylated sites for all ages considered in nuDNA, possibly because mtDNA and methylated groups are more stable inside the mitochondria due to a “double‐protection” as well as a short size (Deiner et al. 2017; Jo, Takao, and Minamoto 2022; Jensen et al. 2021). So far, aging sites detected on the whole genome (WGBS) have either been found exclusively within nuDNA (Qiu et al. 2022; Raddatz et al. 2021; Sun et al. 2021) or have been removed during processing (Meer et al. 2018). Mitochondrial methylation is controversial (Chatterjee, Das, and Chakrabarti 2022), but some evidence indicates a predominance of 6 mA methylation over 5mC methylation (the inverse of the pattern in nuDNA; Hao et al. 2020). Cytosine modifications have typically been used to fit epigenetic clocks, but they might be scarce or even absent from the mtDNA of some taxa (Shao, Han, and Zhou 2023; Sharma, Pasala, and Prakash 2019; Sturm et al. 2023). Decreased 5hmC levels but not 5mC levels (Dzitoyeva, Chen, and Manev 2012) have also been observed for nuclear 5mC sites (Suarez‐Bregua et al. 2021), while higher 6 mA levels within mtDNA have been linked to aging (Sturm et al. 2023). Both 5mC and 6 mA are distributed throughout most of the mtDNA, but we observed apparent non‐random aging site distribution patterns (Figure 3A) that do not necessarily match zones with the highest density and methylation levels for 5mC in the fish *Oreochromis niloticus* (e.g., D‐loop; Nedoluzhko et al. 2021). However, some mtDNA sections containing aging sites (i.e., 12S, 16S, tRNA‐Leu1, ND1, ND2, and COI) have already been associated with age‐related diseases in humans (Bellizzi 2017; Ding et al. 2023). Considering multiple modification types appears promising for epigenetic aging, given that a greater number of aging sites correlates with better accuracy (Tangili et al. 2023). In addition, the count of aging sites (493) detected here exceeds the number used to fit most previous epigenetic clocks for wild animals (Tangili et al. 2023), even though mtDNA represents only 0.003% of the *D. labrax* genome. Targeting the mitogenome in future eDNA epigenetic studies using nanopore sequencing might enable the retrieval of long reads that degrade slowly in the mitochondria (e.g., fragments > 10 kb despite shearing; Figure S5 in Data S3; Yao et al. 2022; Jensen et al. 2021). This approach could be used for bias‐free mapping (e.g., NMUTs, which are mtDNA‐like fragments from nuDNA) or even *de novo* mitogenome assembly if sufficient coverage is attained (Franco‐Sierra and Díaz‐Nieto 2020). As most metabarcodes are located on mtDNA, it could be possible to simultaneously identify species using a multi‐marker approach, while estimating age and condition from eDNA epigenetics, or even genetic population structure by comparing samples from various zones (Ramón‐Laca, Gallego, and Nichols 2023).

## 5 Limitations and Perspectives

The main limitations of this study are its low number of successful time points (i.e., only 6 due to technical issues), the short age range considered (10–24 DPH) and the absence of an independent validation dataset. Considering this, we do not aim to provide a reference epigenetic clock, but we instead propose a set of new tools to explore a new promising research question efficiently. Additionally, an advantage of using eDNA is that this approach resulted in pooling DNA from hundreds of individuals in each vial, meaning that the 376 mtDNA seabass reads used to fit epigenetic clocks likely originated from different individuals. Such number of individuals exceeds the minimum optimal sample size of 134 individuals advised by Mayne, Berry, and Jarman (2021) for tDNA. To more thoroughly validate the potential of eDNA epigenetics as a new age assessment method, further work should focus on various species/stages using larger filtration volumes (e.g., 60 L). Researchers should also aim to predict the age of individuals from different populations/species/environments as a way to assess the universality of their clock. A major challenge of amplification‐free eDNA sequencing will be to obtain sufficient eDNA for detecting aging sites (i.e., sufficiently high coverage; Laine et al. 2023) from marine waters, which typically contain much lower densities of organisms than aquaculture tanks, even though abundances might be very high for many commercial species (e.g., pelagic schoolers). To that end, it might be better to filter larger volumes of water and/or use enrichment methods instead of pooling replicates and shearing samples, notably since nanopore sequencing provides satisfying results even at 6.25% of their recommended input (Heavens et al. 2021). Targeted methylation‐sensitive polymerase chain reaction (PCR) toward previously identified aging regions could be used for very small DNA inputs, but the method still does not offer a single‐nucleotide resolution (Mayne et al. 2020; Qi et al. 2021; Sturm et al. 2023). Enrichment appears more attractive in other cases, as it would make it possible to enrich circular DNA (exonuclease; Ramón‐Laca, Gallego, and Nichols 2023) or organelles (differential centrifugation; Jo et al. 2019) for targeting mtDNA, if their integrity is preserved. It would additionally enable depletion of bacterial DNA (Feehery et al. 2013), which is less methylated than animal DNA and was the main source of contamination in our case (Figure S3 in Data S2). Recently, ONT introduced a new method called adaptive sampling to enrich target DNA during sequencing, which already showed promising results enriching environmental mtDNA using sets a full reference mitogenomes from all mammalians (Frank et al. 2024) or all expected species of parasites and hosts (Kipp et al. 2023).

Another major challenge will be to test if reference epigenetic clocks used later for eDNA epigenetics can be obtained from tDNA of wild individuals, as many species are complicated to maintain in captivity during all life stages, and as captivity might cause methylation changes even if there is no evidence that they affect epigenetic clocks (Tangili et al. 2023). This might not be straightforward, as Zhao, van Bodegom, and Trimbos (2022) observed different methylation patterns between eDNA and tDNA for the same ages. However, at least the tissue, sex, and potential error in training age (if estimated) do not seem to have a large effect on epigenetic clocks (Le Clercq et al. 2023; Mayne, Berry, and Jarman 2023; Tangili et al. 2023). Finally, epigenetic markers other than DNA methylation that affect the expression of genes, such as non‐coding RNA or various chromatin features now detectable with nanopore sequencing (Yue et al. 2022), could also be used for age assessments. In particular, as for eDNA, environmental RNA (eRNA) could be useful for identifying species, age, sex ratio, condition, and stress (Stevens and Parsley 2023). eRNA's ability to discriminate life stages of various amphibian species—both in captivity and in the wild—was recently demonstrated (Parsley and Goldberg 2023). This suggests that, when used in conjunction, eRNA and eDNA epigenetics could have an even greater potential for population management and species conservation. After overcoming technical challenges, eDNA/eRNA surveys might become very efficient methods for rapid, synoptic, and non‐invasive screening of a whole ecosystem's health and biodiversity on the basis of multiple variables, since eDNA sampling is generally much easier than the sampling of individual organisms.

## Author Contributions

**Eliot Ruiz:** conceptualization (lead), formal analysis (lead), methodology (lead), resources (lead), software (lead), visualization (lead), writing – original draft (lead), writing – review and editing (lead). **Fabien Leprieur:** supervision (equal), writing – review and editing (equal). **Gérard Sposito:** resources (equal), supervision (equal). **Martina Lüthi:** methodology (equal), resources (equal), writing – review and editing (equal). **Michel Schmidlin:** methodology (equal), resources (equal), writing – review and editing (equal). **Jacques Panfili:** supervision (equal), writing – review and editing (equal). **Loïc Pellissier:** conceptualization (equal), funding acquisition (equal), writing – review and editing (equal). **Camille Albouy:** conceptualization (equal), funding acquisition (equal), project administration (equal), supervision (equal), writing – review and editing (equal).

## 6 Conflicts of Interest

The authors declare no conflicts of interest.

## Supporting information


Data S1.

## Data Availability

All data and scripts generated during this study are publicly accessible. Codes and raw figures were deposited on Github: https://github.com/ruizeliot/eDNA_epigenetic_aging_seabass_2024. Data were deposited on FigShare: https://doi.org/10.6084/m9.figshare.25466890.
