# Supplementary material for: Environmental DNA Epigenetics Accurately Predicts the Age of Cultured Fish Larvae
Source: Ecol Evol. 2025 Feb 12;15(2):e70645. doi: 10.1002/ece3.70645 (PMC11821287; doi:10.1002/ece3.70645)
Supplement: Supplementary file 1 — Data S1. [file ECE3-15-e70645-s001.pdf]

Supplementary Material

Environmental DNA epigenetics accurately predicts the age of cultured fish larvae

Authors: Eliot Ruiz [1], Gérard Sposito [2], Martina Lüthi [3,4], Michel Schmidlin [3,4], Jacques Panfili [1], Fabien Leprieur [1*], Loïc Pellissier [3,4,*], Camille Albouy [3,4,*]

[1] MARBEC (Marine Biodiversity Exploitation and Conservation), Univ. Montpellier, IRD, IFREMER, CNRS, Montpellier, France

[2] Mediterranean Coastal Environment Station, University of Montpellier, Sète, France

[3] Ecosystems and Landscape Evolution, Institute of Terrestrial Ecosystems, Department of Environmental Systems Science, ETH Zürich, Zürich, Switzerland

[4] Land Change Science Research Unit, Swiss Federal Research Institute WSL, Birmensdorf, Switzerland

* shared senior authorship

Contact information: eliotruiz3@gmail.com

SUPPLEMENT 1 | DNA preparation and sequencing summary

The eDNA weight (ng) obtained after the extraction varied widely between replicates with the same age, with a mean coefficient of variation of 68.9%. To better understand the causes of this variability, we tested the eDNA weight for each replicate against the duration of eDNA shedding (from the introduction of larvae into the sterile water up to the filtration), the approximate density of larvae per experiment, and the volume of water filtered (2 or 3 L). Using weight measurements from hatching for *Dicentrarchus labrax* from Kamacı et al. (2010), we also computed the putative weight of larvae per age class through linear interpolation (*approx* function in R), and multiplied it by the density and the volume to estimate the biomass within each bucket (**Table S1**).

A robust regression (*lmrob* function from the “robustbase” R package; Maechler et al., 2023) revealed that the larval biomass in each bucket was the most significant variable explaining the eDNA weight obtained after the extraction ($t(19) = 4.225,\ p < 0.001,\ r_S = 0.25$), with an increase of 392 ng of eDNA (999 times bootstrapped 95% confidence interval [CI], implemented with the “boot” R package: $[124, 693]$; Canty & Ripley, 2022) obtained after the extraction for each 1 g increase in biomass (**Figure S1.A**). The age was also significant ($t(19) = -4.754,\ p < 0.001,\ r_S = 0.28$), with a decrease of 20 ng per day post-hatch (DPH), but its bootstrapped CI overlapped 0 ($[-46, 0.26]$; **Figure S1.B**). The volume filtered and the duration of eDNA shedding were not significant predictors of the eDNA weight extracted (**Figure S1.C** & **S1.D**).

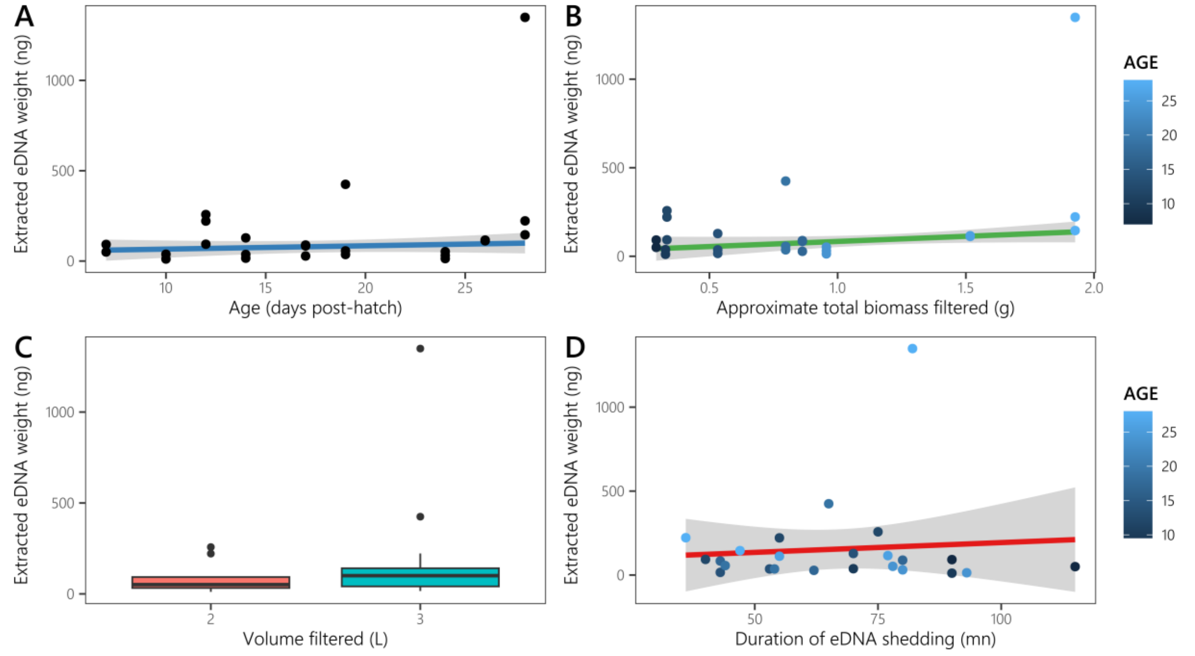

FIGURE S1 | *(A) Robust linear regression of the relationship between extracted eDNA weight per replicate and Dicentrarchus labrax (seabass) larvae age. Effects of (B) the approximated larval biomass, (C) the volume filtered increased from 2L to 3 L from 12 days post-hatch (DPH), and (D) the duration of eDNA shedding on the extracted eDNA weight modelled with a robust linear regression taking into account all three factors, as well as the age (represented by dot shade).*

During the library preparation, a large part of the extracted DNA was lost, notably due to the concentration and shearing steps (**Figure S2.A**; **Table S2**). The mean percentage of loss was 56.3% (SD: 36.9%) for the control samples, and 62.8% (SD: 12.2%) for the test samples, which were pooled together. Despite pooling the three replicates, the DNA input in the MinION was below the minimum

recommended input (400 ng) for the Native Barcoding Kit 24 we used, except for two samples out of nine (**Figure S2.B**). However, two robust regressions revealed that neither the total number of reads ($t(16) = 0.230,\ p = 0.82$) nor the number of 90% assigned seabass reads ($t(16) = -0.192,\ p = 0.85$) could be predicted by the DNA volume added into the MinION (**Figure S2.C** & **S2.D; Table S3**). Including the sum per triplicate for each predictor of the eDNA volume after the extraction (**Figure S1**) also could not predict the total number of reads ($F(4) = 0.240,\ p = 0.90$) or the number of seabass reads ($F(4) = 0.186,\ p = 0.93$) in a parametric regression.

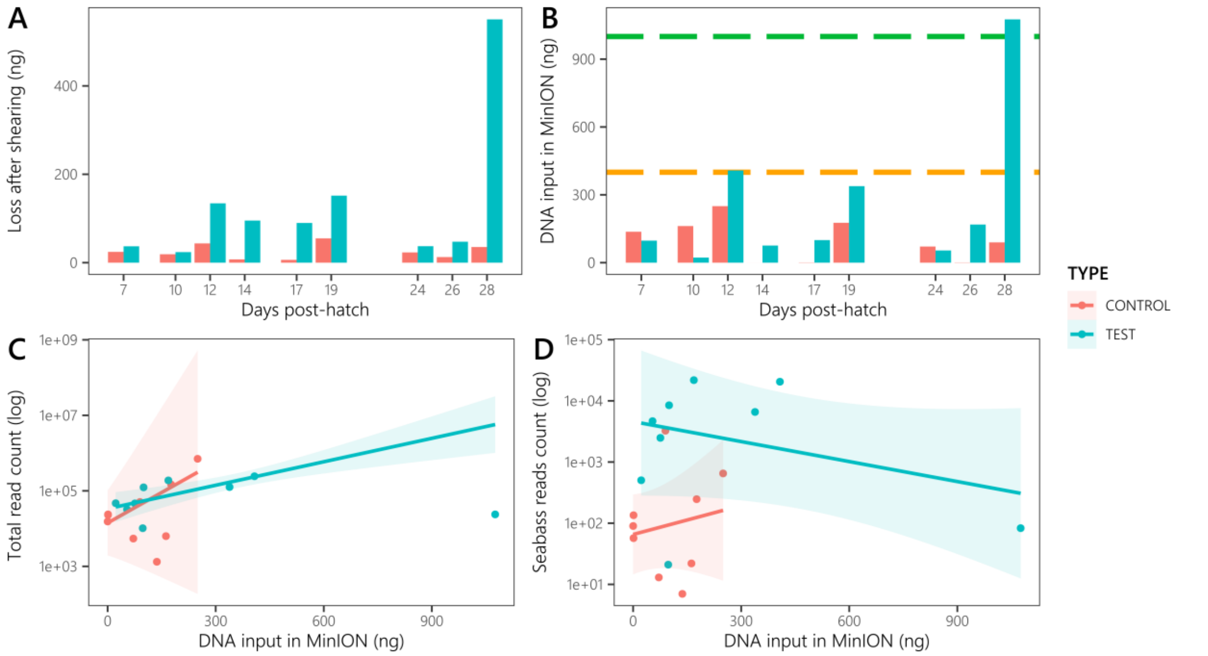

FIGURE S2 | *DNA loss after shearing in control samples and pooled test samples for each experimental stage (A), which conditioned the DNA input in the flow cell (B), which needed to be at least 400 ng (orange dashed line) and ideally above 1000 ng (green dashed line). Relationship between the DNA input and the total number of reads (C), or the number of assigned seabass reads (D), for each sample type (colour), modelled with robust linear regressions.*

AGE (DPH)	DENSITY (ind.L^{-1})	VOLUME (L)	CODE	SAMPLE	DURATION (min)	DNA CONCENTRATION (ng. µL^{-1})	DNA QUANTITY (ng)
**7**	150	2	P1A	R1	60	NA	NA
			P1B	R2	90	0.97	92.2
			P1C	R3	115	0.53	50.4
			C1	C	130	1.79	170.1
**10**	100	2	P2A	R1	30	NA	NA
			P2B	R2	70	0.40	38.0
			P2C	R3	90	0.12	11.4
			C2	C	100	2.01	191.0
**12**	80	2	P3A	R1	40	0.98	93.1
			P3B	R2	55	2.33	221.4
			P3C	R3	75	2.71	257.5
			C3	C	85	3.26	309.7
**14**	80	3	P4A	R1	43	0.17	16.2
			P4B	R2	53	0.38	36.1
			P4C	R3	70	1.35	128.3
			C4	C	79	0.08	7.6
**17**	80	3	P5A	R1	43	0.88	83.6
			P5B	R2	62	0.29	27.6
			P5C	R3	80	0.93	88.4
			C5	C	90	0.08	7.6
**19**	60	3	P6A	R1	44	0.59	56.1
			P6B	R2	54	0.38	36.1
			P6C	R3	65	4.47	424.7
			C6	C	78	2.57	244.2
**24**	60	2	P7A	R1	78	0.55	52.3
			P7B	R2	80	0.33	31.4
			P7C	R3	93	0.14	13.3
			C7	C	70	1.05	99.8
**26**	50	3	P8A	R1	55	1.18	112.1
			P8B	R2	77	1.22	115.9
			P8C	R3	92	NA	NA
			C8	C	50	0.15	14.3
**28**	50	3	P9A	R1	36	2.34	222.3
			P9B	R2	47	1.53	145.4
			P9C	R3	82	14.20	1349
			C9	C	50	1.39	132.1

TABLE S1 | *Extracted eDNA concentration (ng.µL^{-1}) and total quantity (ng) in 95 µL per sample (5 µL for Qubit measurements), categorized as replicates (R) or controls (C), and later grouped per pool (P). Metadata characterizing each experimental stage (i.e., density of larvae and volume filtered) and each sample (i.e., DNA shedding duration) are also presented.*

AGE (DPH)	SAMPLE	BARCODE	BEFORE SHEARING (ng)	SHEARING LOSS (ng)	AFTER SHEARING (ng)
**7**	P1	bar1	134.5	37.0	97.4
	C1	bar10	161.1	24.4	136.7
**10**	P2	bar2	46.3	23.9	22.4
	C2	bar11	180.9	19.0	161.9
**12**	P3	bar3	541.8	134.3	407.6
	C3	bar12	293.4	43.6	249.8
**14**	P4	bar4	170.9	95.3	75.7
	C4	bar13	7.4	7.4	0.1
**17**	P5	bar5	189.6	89.9	99.8
	C5	bar14	7.4	6.3	1.1
**19**	P6	bar6	490.1	151.7	338.4
	C6	bar15	231.3	55.0	176.3
**24**	P7	bar7	91.2	37.3	53.9
	C7	bar16	94.5	23.2	71.3
**26**	P8	bar8	216.0	47.4	168.6
	C8	bar17	13.8	12.7	1.1
**28**	P9	bar9	1626.3	550.5	1075.8
	C9	bar18	125.1	35.4	89.7

TABLE S2 | *Available DNA (ng) in 75µL (volume added to the MinION) before and after the shearing step (DNA input in the MinION in that case).*

AGE (DPH)	SAMPLE	BARCODE	N° TOTAL READS	N° SEABASS READS	N° SEABASS MTDNA
**7**	P1	bar1	10239	21	0
	C1	bar10	1322	7	0
**10**	P2	bar2	46961	503	8
	C2	bar11	6310	22	0
**12**	P3	bar3	243197	20524	92
	C3	bar12	705020	651	6
**14**	P4	bar4	46480	2482	32
	C4	bar13	15416	90	0
**17**	P5	bar5	123698	8465	107
	C5	bar14	23724	135	0
**19**	P6	bar6	126077	6577	47
	C6	bar15	146851	247	0
**24**	P7	bar7	32253	4656	19
	C7	bar16	5439	13	0
**26**	P8	bar8	188774	21779	57
	C8	bar17	22966	57	0
**28**	P9	bar9	23762	83	0
	C9	bar18	50691	3243	8

TABLE S3 | *Number of reads per sample obtained after superior accuracy basecalling without Q-score filtration. Among the total number of reads assigned to each barcode after demultiplexing, reads were assigned to the reference whole genome, or the reference mitogenome for D. labrax using VSEARCH and a 90% minimum similarity threshold.*

SUPPLEMENT 2 | *Ex situ* amplification-free nanopore sequencing considering full genomes

The raw folder generated by the MinKNOW software (*i.e.*, fast basecalling and demultiplexing using GUPPY) was processed so that all the reads in the separate FASTQ files for each barcode that passed the Q-score filter were aggregated into the same FASTA file. As no selection/amplification was carried out during library preparation, the sequenced reads could correspond to any organisms and any positions on their genome, unlike barcoding and metabarcoding. Therefore, assigning all 1.38 million reads to the whole NCBI nucleotide database in a reasonable amount of time would not have been possible with the classic BLAST method. To get an idea of the communities that were sequenced, we first selected a subset of reads relatively close to seabass DNA by assigning all reads to all *Dicentrarchus labrax* sequences available from NCBI on 08/2023 using a 50% minimum similarity threshold in VSEARCH. As running BLASTN on a local database was too slow, even on a CPU server, we split all 21,516 reads in multiple files so that the total number of nucleotides per file did not exceed 1 million, which is the limit for online BLASTN queries. Online queries lasted about 25–30 h per file (parallelized for the seven files), and we reported only the five top hits for each read.

Seabass reads were detected from all test samples except the first (7 DPH) and the last (28 DPH), as well as from five control samples, which were filtered seawater without seabass larvae (12, 17, 19, 26 & 28 DPH). Such contamination of control samples with seabass DNA might originate either from unfiltered eDNA that was present in the seawater pumped near the aquaculture facility, or from airborne eDNA within the facility, since various tanks 10 m apart from the experiment zone contained many *D. labrax* with different ages. The best matches for each read had a mean identity of 98.7% and revealed that even in this subset of reads presenting some similarities with seabass DNA, the latter was not predominant (25.4%), with bacteria representing 29.6%, fungi 23.2%, and viruses 8.4% of the reads (**Figure S3**). Other vertebrates within the subset were 0.3% *Homo sapiens*, 0.8% *Artemia franciscana* (crustacean used to feed seabass larvae), and 0.4% *Sparus aurata* (not aquacultured but present around the pumping zone at sea). All other identified vertebrates seemed to originate from basecalling/identification errors, since three other fish species were closely related to *D. labrax* (but no *D. punctatus*) but were not present in the aquaculture or around the pumping zone (*i.e.*, *Argyrosomus regius, Lateolabrax maculatus,* & *Morone saxatilis*), and two others were simply unrelated, uncultured, tropical species (*i.e.*, *Epinephelus fuscoguttatus* & *Thunnus albacares*).

Although fungi, bacteria, and virus reads probably greatly outnumbered eukaryote reads in the full dataset, our experiment revealed the potential of *ex situ* amplification-free metabarcoding using nanopore sequencing as a non-invasive way to set up identification and aging reference databases if airborne or waterborne eDNA contamination can be avoided. eDNA samples collected *in situ* could then be used for identification and aging if targeted mitogenomes (*i.e.*, aging sites only found there) can be enriched and amplified to alleviate concentration (*i.e.*, very low concentrations of organisms in the ocean) and contamination issues (*i.e.*, DNA of many more organisms are probably present in the ocean).

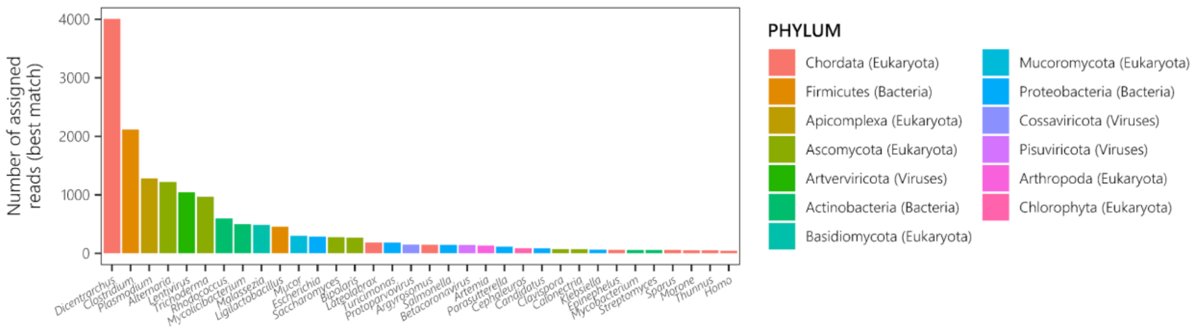

FIGURE S3 | *Number of BLAST best matches per genus (coloured by phylum), considering all genomic regions and all filtered fast basecalled reads sharing more than 50% of similarity with any Dicentrarchus labrax sequences available on NCBI.*

SUPPLEMENT 3 | *Ex situ* amplification-free nanopore sequencing considering only mitogenomes

Contamination of control samples with *Dicentrarchus labrax* eDNA was detected using all reads, which raised the question of whether all aging sites were all from individuals of the same known age. We therefore replicated the analysis but against a database containing only full reference mitogenomes, since aging sites were only found on mitochondrial reads. We downloaded the RefSeq database from NCBI on 08/2023 (https://ftp.ncbi.nlm.nih.gov/refseq/release/mitochondrion/); it contains full and curated mitochondrial genomes from 14,665 eukaryote species from 30 different phyla. To avoid potential basecalling biases affecting the subset of full genomes that were fast basecalled (**Supplement 2**), we assigned all superior basecalled reads with Dorado to the 1 kb chunked RefSeq mitogenomes, using a minimum similarity threshold of 70% in VSEARCH.

Following the guidelines proposed by Ruiz *et al.* (*in prep*) for the optimal gaps to delineate genera/species based on multiple mitochondrial genes and fish species, we considered only reads assigned to genera with ≥90% similarity (675 eDNA reads), and to species with ≥98% similarity (327 eDNA reads). Similarly to the identification of eukaryotes using the subset of full genomes, fungi mitogenomic fragments (43.6%) were more abundant than *Dicentrarchus* (28.6%), *Artemia* (10.5%), and *Homo* (0.1%) reads when considering similarities ≥90% (**Figure S4.A**).

Although no other fish species were identified with a similarity ≥98% (**Figure S4.B**), as for the fast basecalling subset, three Chordate identifications appeared to be basecalling errors and/or incomplete reference database biases, as they were not native and not cultured (*i.e.*, the snake *Elaphe dione* and the bird *Arborophila rufogularis*). On the contrary, the superior basecalling was precise enough to discriminate between the two *Artemia* species for similarities >98%, since it exceeded the similarity of 97.5% between the two reference mitogenomes. As both *Artemia* species were present in the Thau lagoon (Amat et al., 2005) and in seabass larvae faeces, it is not possible to conclude that replacing three times 85% of the seawater from the aquaculture tank with filtered seawater was inefficient in removing all eDNA. However, it seems most probable since the proportions of reads from both species (*i.e.*, *Artemia franciscana* = 89% & *Artemia salina* = 11%; **Figure S4.B**) matched their proportion in the food of seabass larvae throughout the experiment (*i.e.*, mean of 89% for *Artemia franciscana* since the proportion was switched from 100% to 80% at 15 DPH).

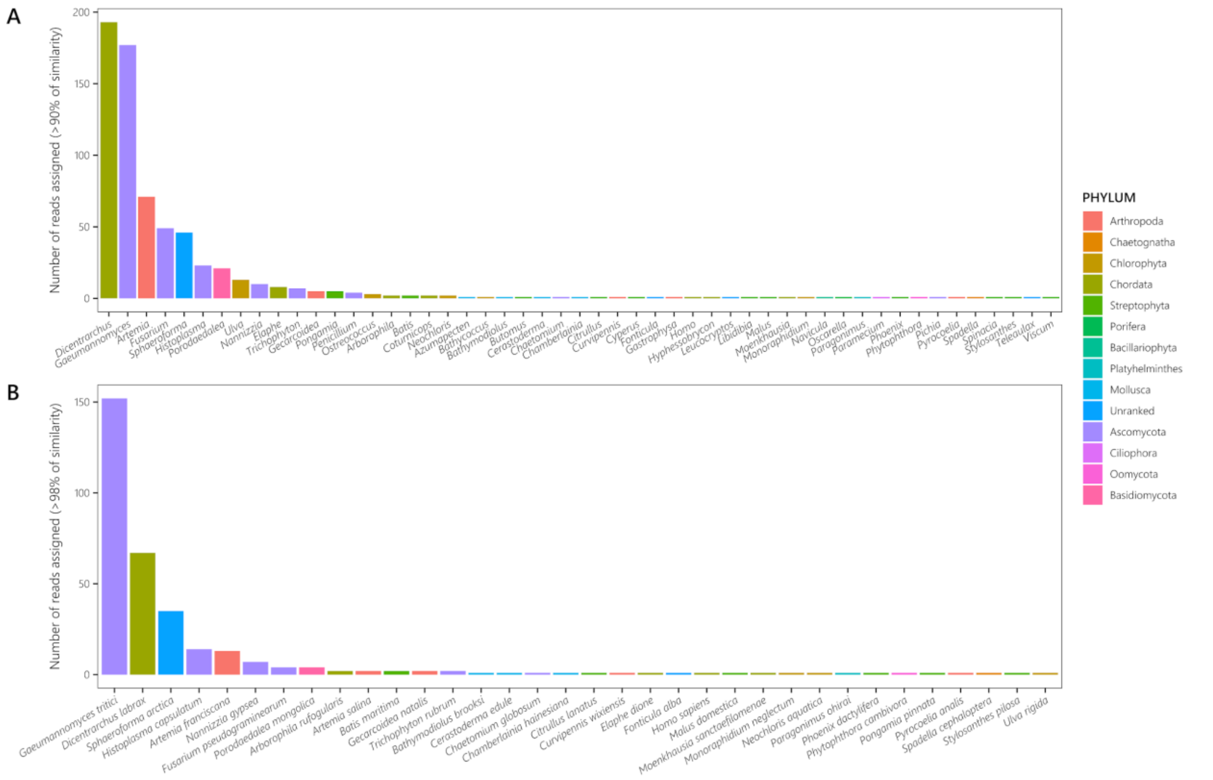

***FIGURE S4** | Number of unfiltered superior basecalled reads reliably assigned by VSEARCH to a RefSeq mitogenome (coloured by phylum), shown (A) per genus and (B) per species.*

Considering all reads assigned to RefSeq mitogenomes (5,903 eDNA reads), another advantage of *ex situ* amplification-free metabarcoding using nanopore sequencing appears to be the length of reads sequenced, as they were on average longer than 1.3 kb (**Figure S5.A**). Despite the potentially important degradation of eDNA associated with a shearing step during library preparation, we were even able to sequence single reads covering 50–100% of their respective reference genome (**Figure S5.B**). *Dicentrarchus labrax* reads had a similar mean length (1.4 kb), and 20 reads exceeding 5 kb were even found in the test samples, with a coverage of >50% (*i.e.*, >9,126 bp) of the reference mitogenome ensured by only four of them (**Figure S5.C**). Importantly, coverage values >100% probably result from assignation to the genome of another species, due to the relatively small size of the RefSeq database and the low minimum similarity threshold used (70%).

Finally, mitochondrial fragments assigned to the reference *D. labrax* mitogenome (no reads assigned to *D. punctatus* even if in the RefSeq database) with a minimum of 70% identity were found only in two control samples, unlike for the full genome subset fast basecalled with GUPPY (**Supplement 2**). The mean similarity to the reference *D. labrax* mitogenome being higher for control samples (92.8%) than for test samples (87.9%), and reads being slightly longer on average (1.7 kb vs 1.4 kb), these two identifications of *D. labrax* eDNA in the sterile water of control buckets seems to be relatively robust (**Figure S5.C**). In our setup, eDNA contamination from individuals with different ages would probably be anecdotical compared with eDNA fragments shed by individuals of known age, as low concentrations of contaminant eDNA have lower probabilities of passing through a nanopore compared with more abundant eDNA fragments. Nevertheless, such potential bias should not be ignored in further studies (*e.g.*, to achieve greater limitation of waterborne/airborne contamination).

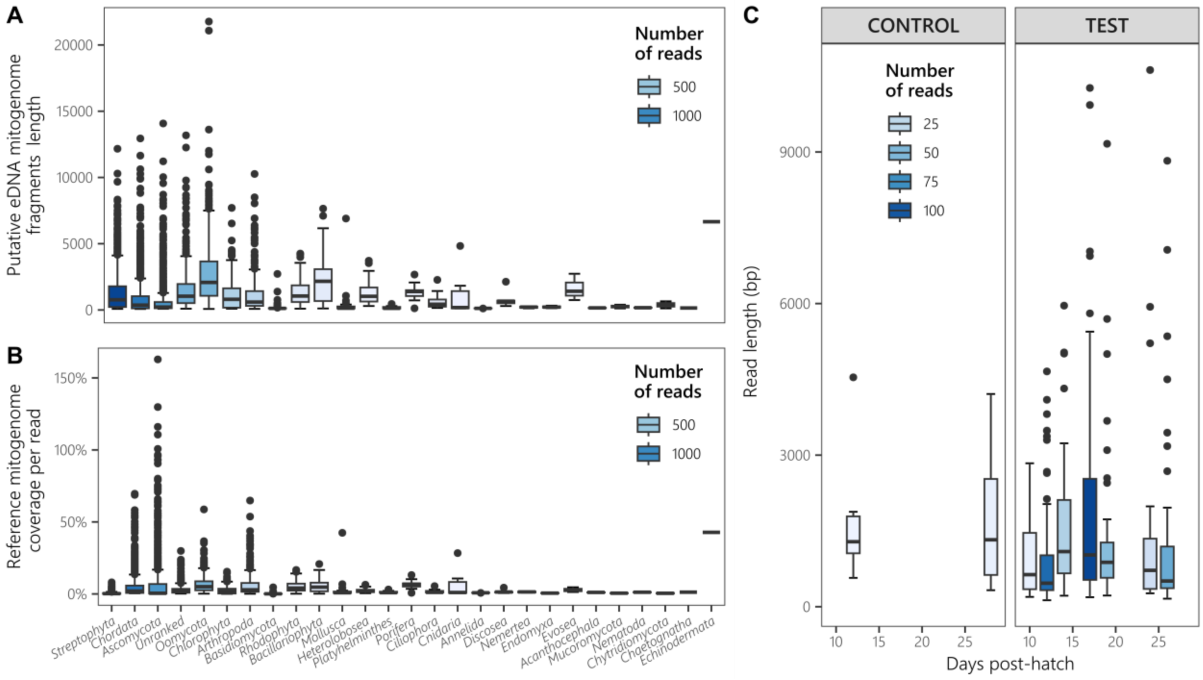

***FIGURE S5** | Putative mitogenomic read (i.e., similarity to a RefSeq mitogenome ≥70%) numbers (boxplot shades) per identified phylum (sorted from left to right), as well as their length (A) and coverage of their assigned RefSeq (B). The length of putative mitogenomic reads assigned to Dicentrarchus labrax is also represented in the same way for each control and pooled test sample (C).*

SUPPLEMENT 4 | Details about read accuracy and identity

The operations “=” and “X”, corresponding to the number of matches and mismatches, respectively, to the reference in the alignment, were empty in the CIGAR string of BAM files outputted by the Dorado aligner. As a result, it was not possible to directly use the simple formulas for the read accuracy and read identity (*e.g.*, https://labs.epi2me.io/quality-scores/).

$$Read\ identity = 100 \times \frac{N_{matches}}{N_{matches} + N_{mismatches}}$$

$$Read\ accuracy = 100 \times \frac{N_{matches}}{N_{matches} + N_{mismatches} + N_{deletions} + N_{insertions}}$$

However, by using the number of substitutions obtained by subtracting the edit distance (“NM” tag) from the number of insertions (“I” operation) and deletions (“D” operation), it was still possible to compute these two metrics as in the *stats_from_aligned_read* function from the “pomoxis” Python package from ONT (https://github.com/nanoporetech/pomoxis/blob/master/pomoxis/stats_from_bam.py). Indeed, as the number of aligned nucleotides (“M” operation) corresponds to the number of matches and mismatches, the number of strict matches can be calculated by simply subtracting the number of substitutions. Therefore, the read identity definition is now:

$$Read\ identity = 100 \times \frac{N_{aligned} - N_{substitutions}}{N_{aligned}}$$

Similarly, the read accuracy can be derived by adding the number of gaps to the number of aligned nucleotides, which gives the alignment length. Unlike “pomoxis”, which simply sums the number of nucleotides aligned to the number of insertions and deletions, we chose to compute the alignment length as the sum of the number of nucleotides aligned with the maximum between the number of insertions and deletions. Indeed, this is the real maximum size of the alignment with respect to the sequence or to the reference (*i.e.*, equivalent of L_{max} in the Supplementary Information of Beyter et al., 2021).

$$Read\ accuracy = 100 \times \frac{N_{aligned} - N_{substitutions}}{L_{max}}$$

The read identity is a better metric of the basecalling accuracy when the real distance between the reference genome and the sequences (*i.e.*, intraspecific variation) is unknown, as basecalling errors should not result in gap additions in alignments. However, the read accuracy is often referred to as read identity and seems to be the metric of choice no matter the context (*e.g.*, the read identity is equivalent to the “gap-excluded identity”, which is not implemented in minimap2: https://lh3.github.io/2018/11/25/on-the-definition-of-sequence-identity). Moreover, different formulas are used for the two metrics (*e.g.*, Beyter et al., 2021; Gleeson et al., 2022; Zhang et al., 2020).

Apart from computing the read accuracy and the read identity from mappings on the full genome (**Figure 2**), we calculated these metrics from mappings on the reference mitogenome, as aging sites (*i.e.*, differentially methylated across ages) were only detected there (**Figure S6**). The medians were only slightly different between the two types of mappings, even though the density functions were more light-tailed for the mitogenomes, meaning there were considerably fewer values with a read accuracy/identity <95% (**Figure S6**).

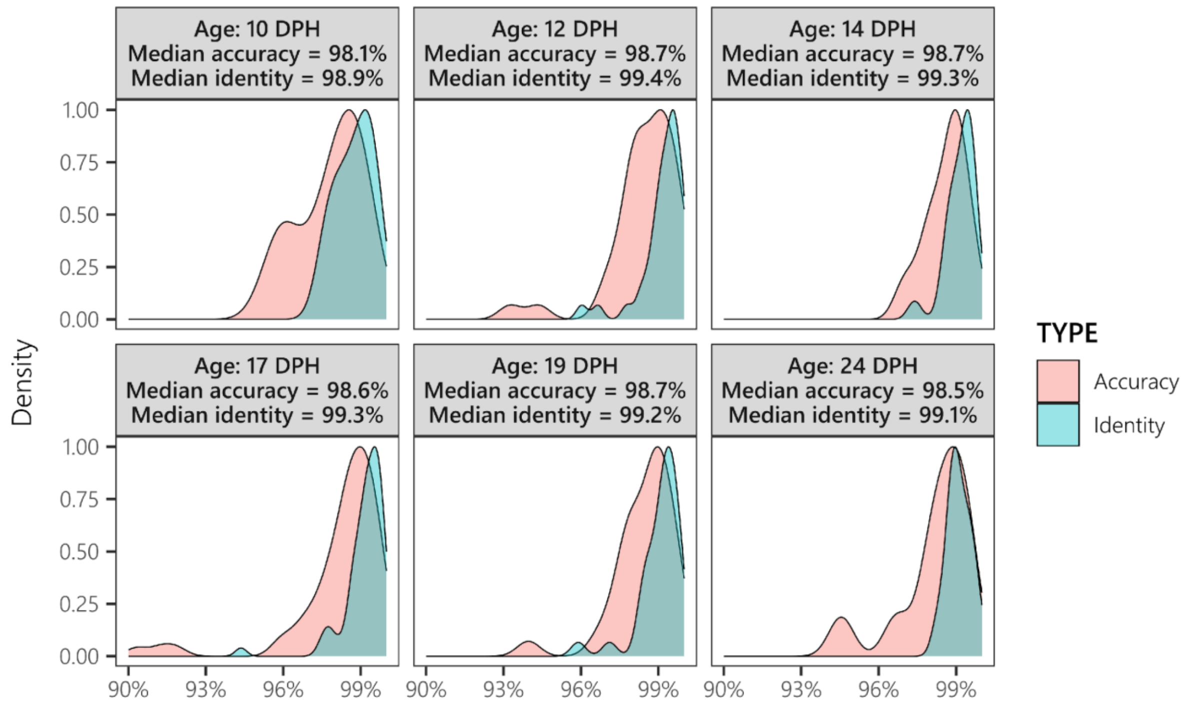

***FIGURE S6** | Density functions of the read accuracy (red) and the read identity (blue) per sample, computed from mappings of Dicentrarchus labrax reads identified with VSEARCH on the reference D. labrax mitogenome, for comparison with the mappings on the reference whole genome (**Figure 2**).*

In addition, we tried to determine the influence of various variables on the read identity in both types of mappings (**Figure S7**). No matter the type of mapping, robust linear regressions (implemented with the *rlm* function from the "MASS" R package since *lmrob* did not converge; Venables & Ripley, 2002) indicated that all predictors were significant ($p < 0.001$), with the most strongly correlated being the read accuracy (mitogenome: $r_S = 0.90$; full genome: $r_S = 0.81$). The unaligned read length was the most negatively correlated predictor for both types of mappings, although the Spearman correlation coefficients were low (mitogenome: $r_S = -0.18$; full genome: $r_S = -0.09$). The number of soft clippings ("S" operation in the CIGAR string), which corresponds to the number of bases at each extremity of a read that do not align well and are therefore not included in the alignment, was also not negatively correlated (mitogenome: $r_S = -0.10$; full genome: $r_S = -0.08$). The other negatively correlated variable (mitogenome: $r_S = -0.14$; full genome: $r_S = -0.01$) was the minimap2 highest alignment score ("AS" tag), which we standardized to between 0 and 1 so that these limits represented the scores of the worst- and the best-aligned reads, respectively.

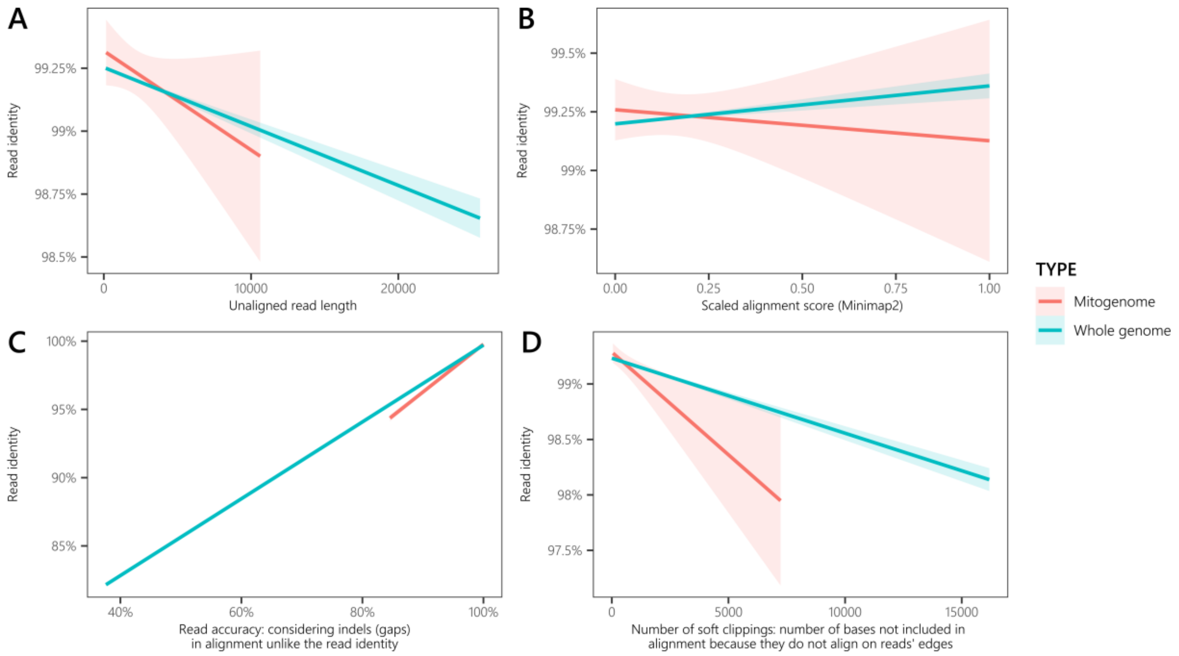

FIGURE S7 *| Relationships between the read identity and all potential predictors included in the robust linear regression, visualized to check their relative effects per type of mapping (colour).*

SUPPLEMENT 5 | Methylated site statistics, coverage, and frequency

Methylation calling summaries per mapped sites aggregated under the BED file format by the program modbam2bed contain various statistics. First, the coverage per position is computed from all mapped nucleotides or gaps, no matter if they are substitutions, deletions (inserted base not counted), or ambiguous cases.

$$Coverage = N_{modified} + N_{unmodified} + N_{unanalyzed} + N_{alternative} + N_{filtered} + N_{substitutions} + N_{deletions}$$

These ambiguous cases can take three forms: a base can be filtered when its modification probability (internally computed) falls below the default threshold of 0.66 ($N_{filtered}$), the targeted modification type (*e.g.*, 5mC) can be another modification type (*e.g.*, 5hmC) detected by the trained model ($N_{alternative}$), or the base might not have been successfully analysed by the methylation calling algorithm ($N_{unanalyzed}$). The methylation level corresponds to the ratio of the number of modified bases with the targeted modification type to the number of other bases matching in the alignment, successfully analysed and unfiltered by the methylation calling algorithm.

$$Methylation\ level = 100 \times \frac{N_{modified}}{N_{modified} + N_{unmodified} + N_{alternative}}$$

The modbam2bed score represents the ratio between unambiguous and ambiguous bases for each position, which can be interpreted as a measure of the reliability of each methylation level, as the numerator excludes the number of alternative modifications, taking into account any risk of confusion between modification types.

$$Reliability\ score = 1000 \times \frac{N_{modified} + N_{unmodified}}{Coverage}$$

For each barcode, we further summarized the statistics for each mapped position in the BED file. We chose to name all mapped adenosines and cytosines "candidate" sites (**Figure S8.A**), as they are the only nucleotides on which it is currently possible to detect methylation with nanopore algorithms. We counted the number of sites that had zero and non-zero coverage ("covered"), to further compute the mean and maximum coverage per barcode for "covered" sites and compare them with the coverage of aging sites (**Figure S9**). We compared the number of sites without unambiguous bases (*i.e.*, "covered" sites with a score of 0) with the number of candidate sites to better understand the extent of ambiguity per barcode (**Figure S8.B**). Then, we enumerated the number of "reliable" methylated sites (**Figure S8.D**), which were defined as those with a methylation level ("methylated") and its associated reliability score ("reliable") different from 0 (*i.e.*, at least one methylated unambiguous nucleotide per position). We compared the number of "reliable" methylated sites with the number of "reliable" candidate sites to check the proportion of methylated sites on the same basis (**Figure S8.E**). Finally, we estimated the number of adenosines and cytosines on both strands of the reference genome (*i.e.*, even unmapped bases) based on the G/C content (G/C: 40%, A/T: 60%) and on the size of the genome (695,892,153 bp), which allowed us to obtain the proportion of "covered" candidate sites (**Figure S8.C**) and of "reliable" methylated sites (**Figure S8.F**) out of the total number of reference bases.

Overall, the number of candidate sites revealed that samples collected at 7 DPH contained too little DNA for any further investigations, notably due to a missing replicate. Conversely, the samples collected at 26 DPH had the largest number of candidate sites, while the samples at 28 DPH were very similar to the ones collected at 7 DPH, probably since the two types of samples were mixed during library preparation (**Figure S8.A**). When only samples from 10 to 24 DPH were considered, a mean of

4.4 and 6.5 million candidate sites were found for modC and modA modifications, respectively, which covered on average 1.58% of the reference bases for both types of nucleotides per barcode. As the proportion of sites represented only by ambiguous bases remained low (*i.e.*, modC = 0.74%; modA = 2.72%), the methylation level of most candidate sites could be assessed, which yielded an average of 142,294 (modC) and 99,187 sites (modA) for samples within the ages of interest (*i.e.*, 10–24 DPH). On average, reliable methylated sites represented 0.80% (modC) and 0.79% (modA) of reliable candidate sites, as well as 0.05% and 0.02% of the reference bases (*i.e.*, difference in A/C content), respectively. Among the modification types of the cytosines detected by the latest Dorado models, 5mC modifications were far more common than 5hmC or other unidentified cytosine types (**Figure S8.D**). Surprisingly, 6mA modifications were represented almost as much as 5mC modifications, with an average of 99,187 6mA sites and 138,915 5mC sites per barcode.

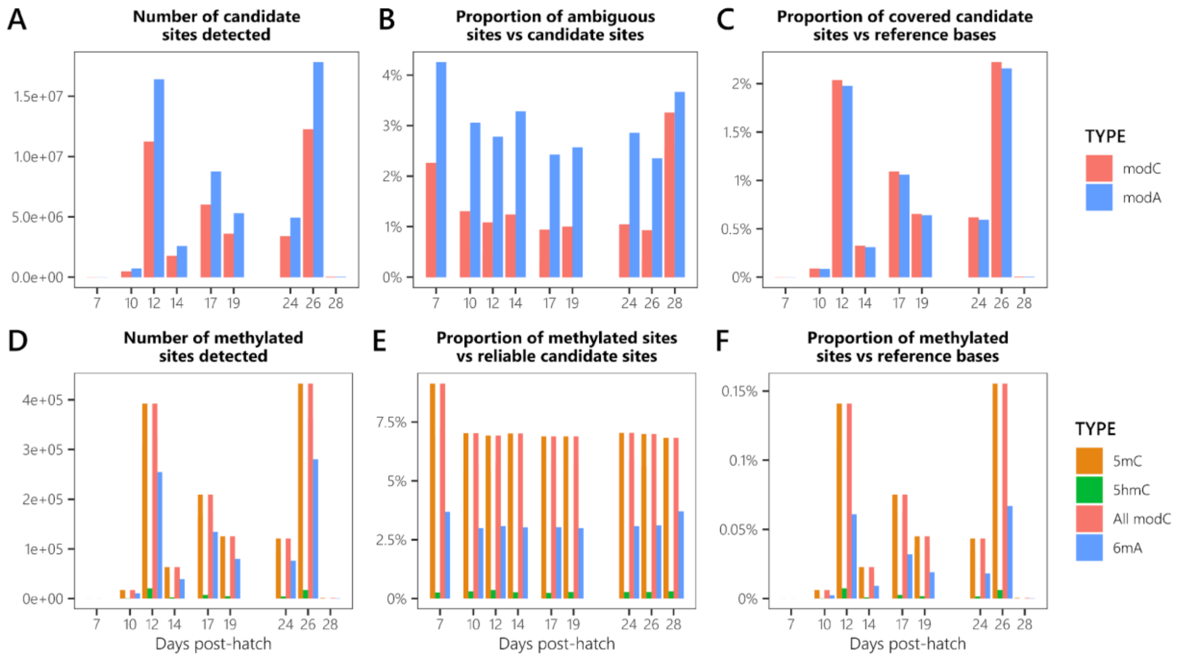

FIGURE S8 | *Representation of the methylation calling main summary statistics per experimental stage for candidate sites for each modified nucleotide (A–C) or for methylation sites per modification type (D–F).*

A potential coverage bias could hamper the search of aging sites from a limited amount of DNA material, as differentially methylated sites across ages had significantly higher coverage than all candidate sites ($F(1,36) = 44.55, p < 0.001$), with a large effect size ($\eta_p^2 = 0.60$) in an aligned rank transformed analysis of variance (ART ANOVA; *i.e.*, non-parametric two-way ANOVA implemented with the R package "ARTool"; Wobbrock et al., 2011). This relationship between coverage and aging sites probably explains why all aging sites were found on the mitochondrial genome, which had mean coverage per candidate site of 3.00X versus 1.01X for all candidate sites, while it represented only 0.06% of the mapped sites (**Figure S9**). Conversely, there was no significant difference in coverage between modification types in the same ART ANOVA, and the interaction between modification type (*i.e.*, 5mC, 5hmC & 6mA) and site type (*i.e.*, candidate or aging site) was not significant. Indeed, all three types had very similar mean coverages (5mC = 3.0X, 5hmC = 3.2X, & 6mA = 3.0X), mean reliability scores (5mC = 964, 5hmC = 914, & 6mA = 950), and mean methylation levels per barcode (5mC = 6.8%, 5hmC = 7.6%, & 6mA = 8.4%).

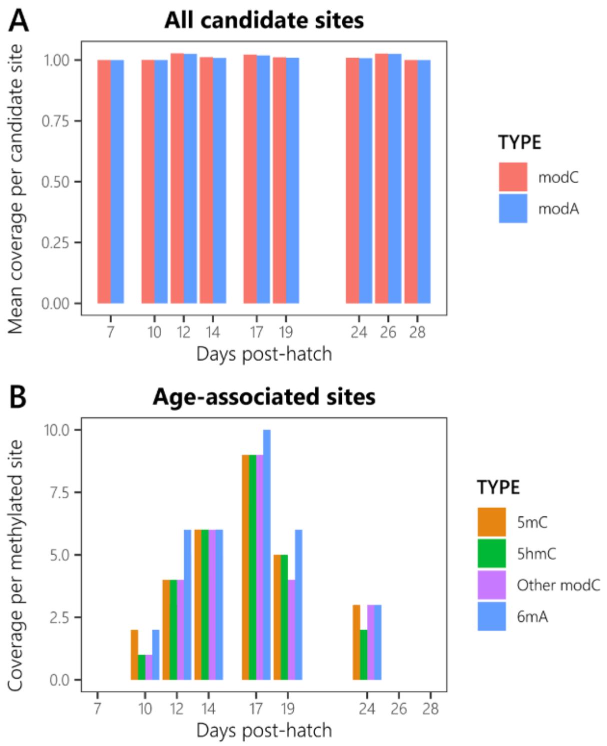

FIGURE S9 | *Mean coverage per experimental stage of candidate sites per modified nucleotide (A) and of methylation sites per modification type (B).*

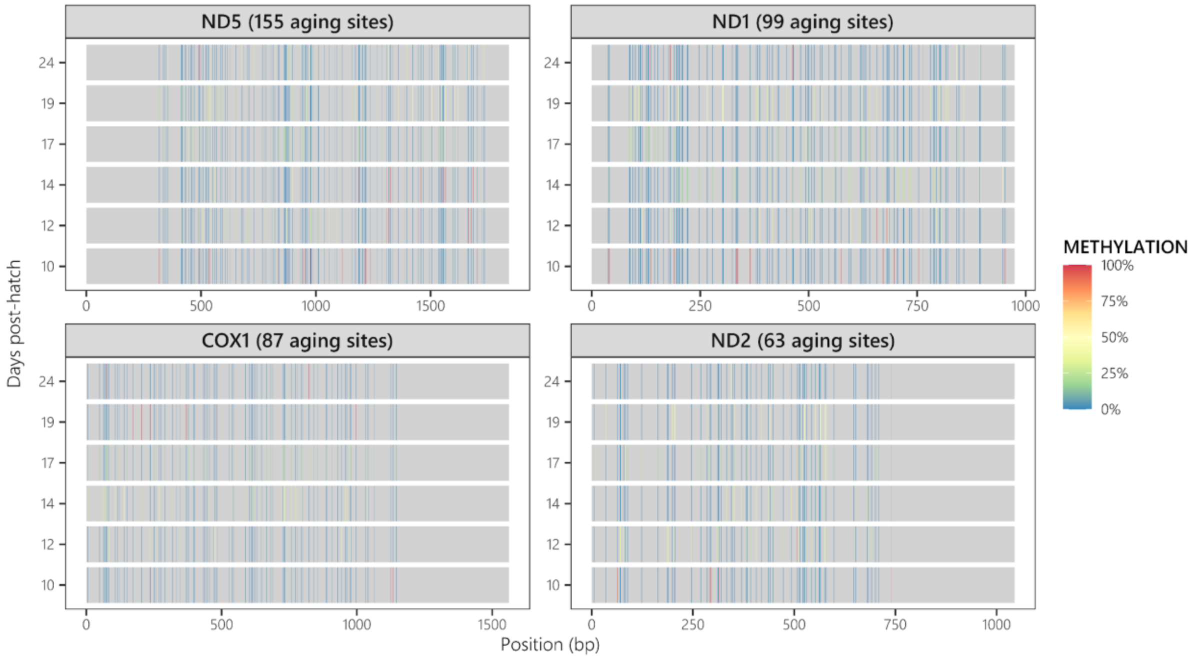

FIGURE S10 | *Representation of the evolution of methylation levels across ages (rows) per aging site (vertical bars) for each codon-organized gene. Positions of aging sites on the grey rectangle (full gene) were obtained from mappings on the reference Dicentrarchus labrax mitogenome.*

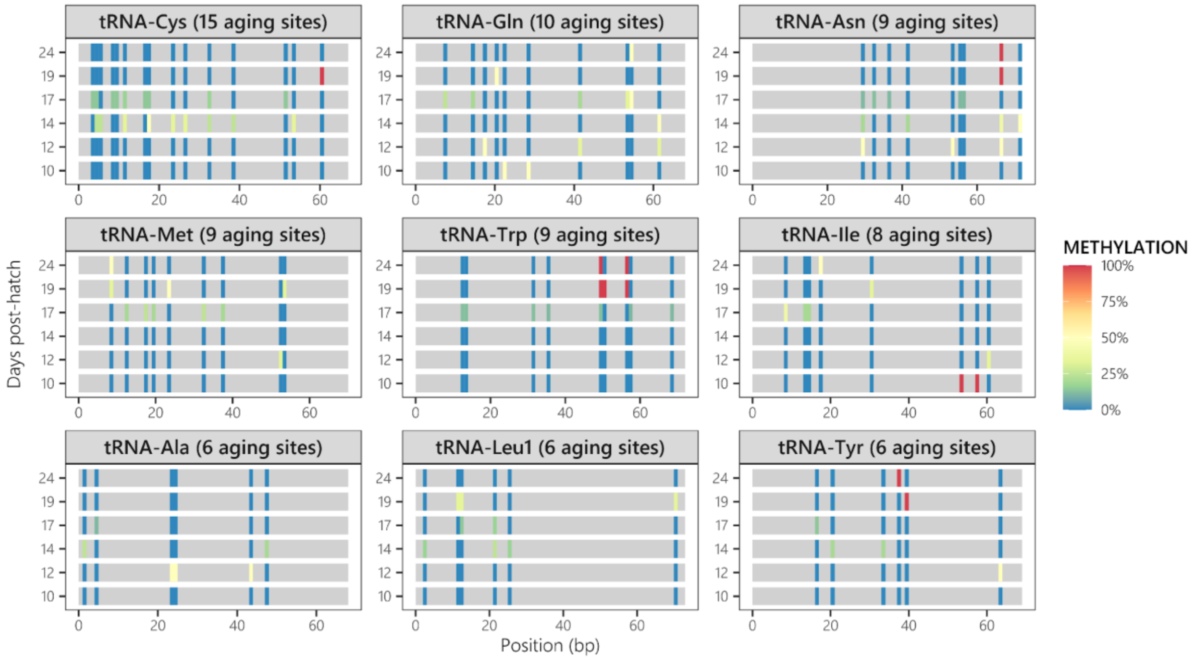

FIGURE S11 | *Representation of the evolution of methylation levels across ages (rows) per aging site (vertical bars) for each tRNA gene. Positions of aging sites on the grey rectangle (full gene) were obtained from mappings on the reference Dicentrarchus labrax mitogenome.*

SUPPLEMENT 6 | Grouping factors and best epigenetic clock selection

Epigenetic clocks were fitted not only on the basis of the methylation level matrix, as in most other studies, but also using various grouping factors (**Figure 5**). Apart from qualitative factors (*i.e.*, gene and modification type), we also included the mean reliability scores and the mean coverage across samples within the ages of interest (*i.e.*, 10–24 DPH; **Figure S12**). A PERMANOVA (*adonis2* function from the R package "vegan" with 999 permutations; Oksanen et al., 2022) revealed that all three factors except the modification type had a significant effect on methylation levels (*i.e.*, Euclidian distance between aging sites), although the two significant interaction terms included the modification type (*i.e.*, interaction with the gene and interaction with the three other factors). However, standardized effect sizes were very small to small for all significant terms, except for medium effects in the case of the gene ($\omega_{p^2} = 0.11$). Although we could only consider the mean reliability and the mean coverage when fitting epigenetic clocks, we used a Mantel test (999 permutations with the *mantel* function from "vegan") to compare the methylation level, reliability, and coverage Euclidian distance matrices among sites. Both variables were significantly linked with methylation levels per site, but their respective effects were small for the reliability ($r = 0.06, p < 0.001$) and very small for the coverage ($r = 0.13, p < 0.001$), and matrices for these two variables were not significantly linked ($r = 0.06, p < 0.001$).

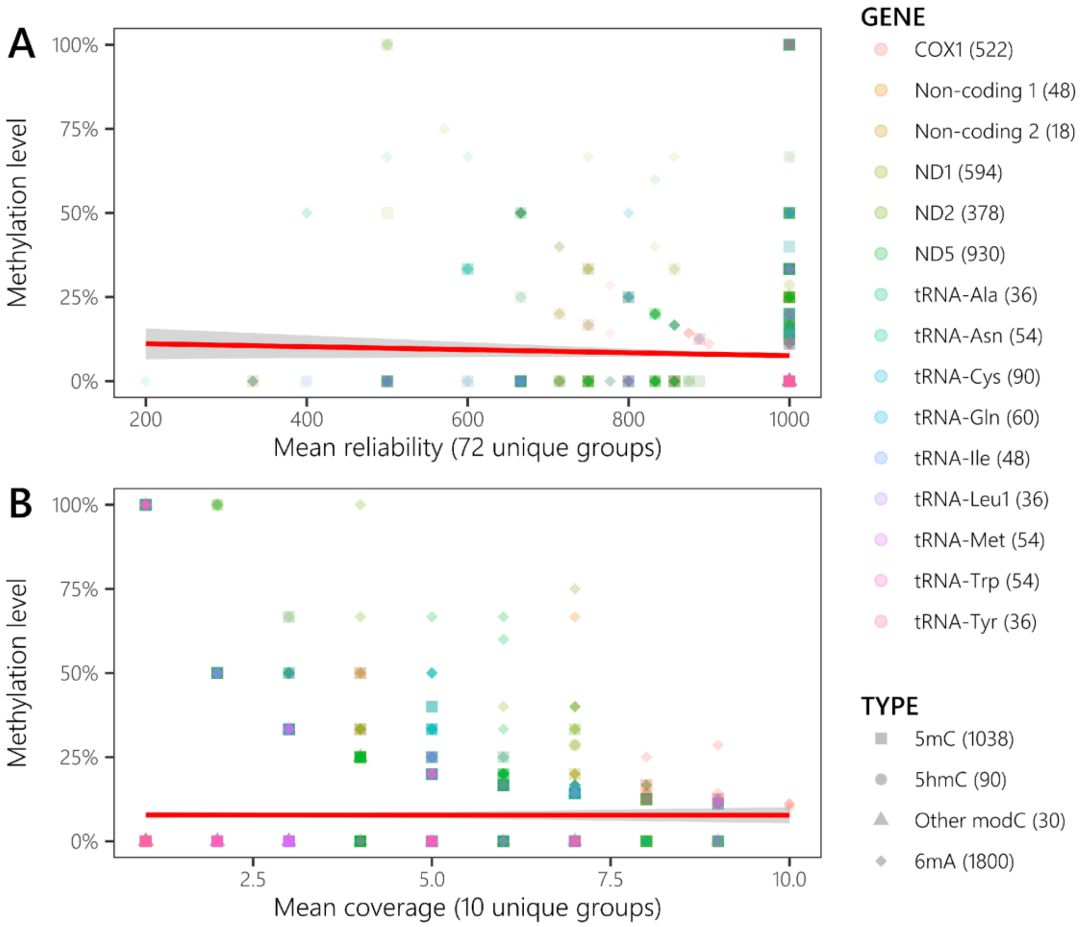

FIGURE S12 *| Representation of variables used as grouping factors for fitting grouped epigenetic clocks. Linear regressions of the two quantitative grouping factors –the mean reliability (A) and the mean coverage (B) across ages – with the methylation level for each age are shown as red lines. The intensity of the colours represents the number of sites sharing the same characteristics.*

To graphically demonstrate the relative performance of epigenetic clocks fitted across values of the parameter α and across bootstrap iterations, as well as the decision process to select the best epigenetic clock, we focused only on the best clocks fitted on the full dataset or datasets comprising only modC or modA. First, **Figure S13** shows that subjectively choosing the value of the parameter α, as done in many studies, can be misleading because optimal penalty factors can vary between subsets of the same dataset and depend on the criteria chosen to select the best clocks. First, this approach could also be confusing because very similar results can be achieved with very different values of α, and conversely (*e.g.*, middle top panel in **Figure S13**). Second, **Figure S13** leads to an understanding of the stochasticity of epigenetic clock fitting because both types of MAE can greatly vary among bootstrap iterations for the same penalty factor α, which would yield unstable results if directly using the *cv.grpnet* (left top panel in **Figure S13**) or the *cv.glmnet* (middle and right top panels in **Figure S13**) functions. Third, such plots validate that 10 bootstrap iterations were sufficient in this case to find optimal clocks, as cross-validated MAE reached a minimum at the centre of an inverted bell curve (left and middle top panels in **Figure S13**) or at one extreme of a linear curve (right top panel in **Figure S13**). Lastly, **Figure S13** demonstrates that clocks with the lowest cross-validated MAE are not necessarily the ones with the lowest final MAE for predictions on the full dataset, which is why we chose to use both metrics for the selection of optimal epigenetic clocks.

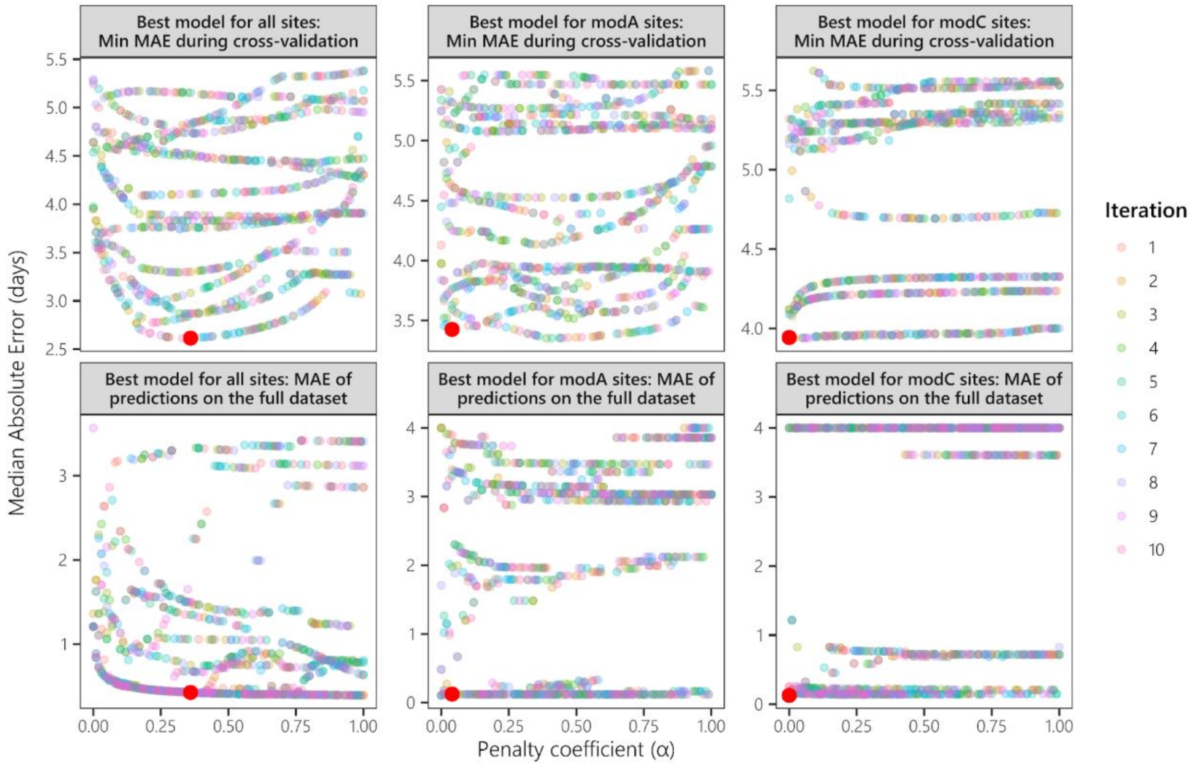

***FIGURE S13** | Representation of performance metrics (rows) of all epigenetic clocks that were fitted using the same combination of grouping factors as the best epigenetic clock (**Figures 6** & **7**) obtained per training dataset (columns). The* epigenetic_clock_bootstrap *function fits epigenetic clocks for every 0.01 increments of α between 0 and 1 (default), which is repeated 10 times (default) in parallel and therefore yields 1010 models whose cross-validated and final median absolute errors (MAEs) are represented by transparent dots. The best epigenetic clock (red opaque dots) is then selected as the one with the best scores for both performance metrics (equal weights).*

SUPPLEMENT 7 | Comparison of various basecalling and assignation methods

To determine the influence of the initial filtering per Q-score (*i.e.*, Q > or ≤ 8) of the basecalling model (*i.e.*, fast or superior accuracy) and of the reference genome chunking (*i.e.*, in 1 kb or 10 kb fragments) on the number of *D. labrax* reads assigned, which could impact the number of aging sites detected, we replicated analyses in this paper three additional times with various combinations of these parameters. The mean number of reads across test samples assigned to *D. labrax* was multiplied by five between the worst (*i.e.*, filtered fast basecalled & 10 kb assigned) and the best analysis (*i.e.*, unfiltered superior basecalled & 1 kb assigned). The average ratios between the mean number of reads across control and test samples obtained from assignations on a 1 kb versus 10 kb chunked reference genome (1.85) were very similar to the average ratios obtained the same way for filtered fast versus unfiltered superior basecalled reads (2.37), showing that choosing all these parameters adequately is crucial to obtain the best results (**Figure S14.A**). However, the obtained unfiltered superior basecalled reads were on average 562 bp (test samples) and 1028 bp (control samples) smaller than filtered fast basecalled reads when they were assigned on 1 kb chunks, which yielded, in general, reads longer than 10 kb chunks (**Figure S14.B**).

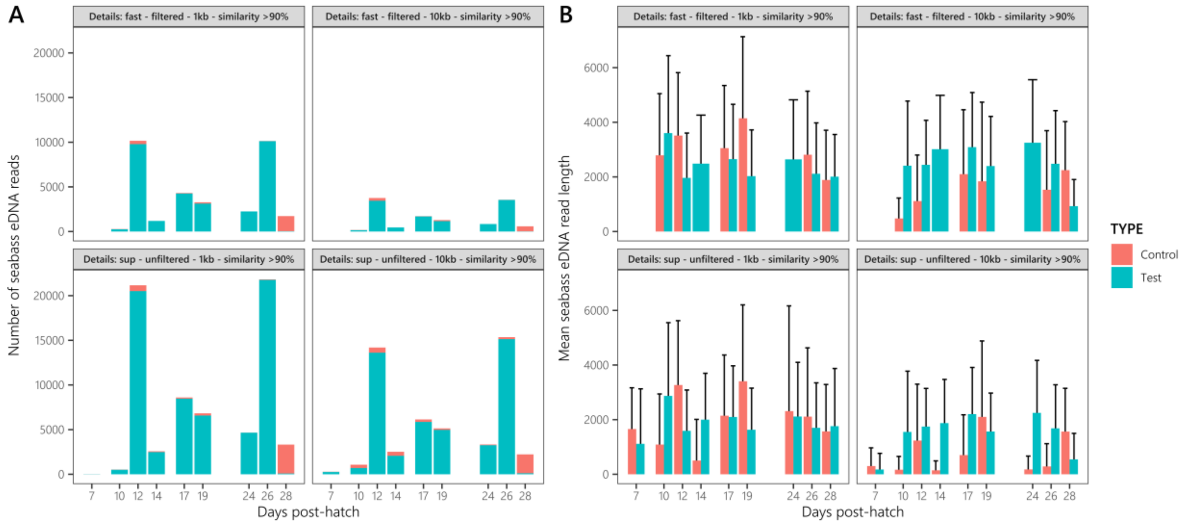

***FIGURE S14** | Comparison of the number of assigned Dicentrarchus labrax reads (A) and of their length (B) among replicated analyses using basecalling, filtering and chunking parameters other than the ones chosen in the main analysis (bottom left panel).*

Detection of aging sites was only conducted for unfiltered superior basecalled reads, to compare their relative effects of 1 kb and 10 kb on the number identified. As the smallest number of assigned reads was obtained when a 10 kb chunked reference genome (which also greatly slows VSEARCH) was used, the mean proportion of candidate and methylated sites on the number of reference bases (0.60% and 0.03% respectively) was smaller than for the 1 kb dataset (0.77% and 0.04%, respectively). The combination of a smaller number of methylated sites and a lower coverage of the mitogenome resulted in the identification of almost twice as fewer aging sites for the 10 kb dataset (296 vs 493), which had a lower mean coverage across samples (2.52X vs 3.03X) but a slightly higher mean reliability score (960 vs 954). Proportions between modification types were similar between the two datasets, but aging sites were detected on five additional tRNA for the 1 kb dataset (**Figure 3**), and the proportion of aging sites on each gene differed between datasets (especially for COX1; **Figure S15**).

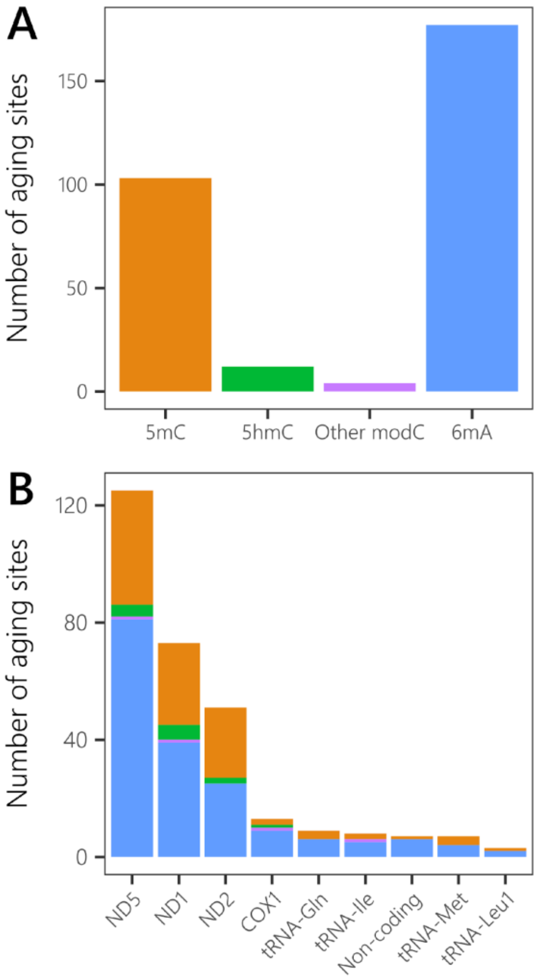

FIGURE S15 | *Number of aging sites detected per modification type (A) and per mitochondrial region (B) using the 10 kb unfiltered superior basecalled dataset, for comparison with its 1 kb counterpart used in the main analysis (**Figure 3**).*

Finally, using the 1kb dataset, we compared the number of aging sites detected from the outputs of various methylation-calling submodels that can identify the same modification type. The 5mC submodel led to the detection of 168 modC aging sites not identified by the 5mCG_5hmCG submodel, but the latter also identified 15 unique modC aging sites. Similarly, the 5mC submodel detected 175 unique 5mC aging sites, while the 5mCG_5hmCG submodel only detected 1 unique 5mC aging site. This result highlights that the 5mC submodel successfully detects 5mC in non-CpG contexts, even though its broader training makes it less accurate for finding all 5mC aging sites in CpG contexts than the 5mCG_5hmCG submodel. Other modC aging sites categorized neither as 5mC nor as 5hmC were also only detected by the 5mCG_5hmCG submodel, which could be a model training problem or indicate interferences in the signal by modifications in other cytosines (*i.e.*, 4mC, 5fC, and 5caC), also detectable with nanopore sequencing (Tourancheau et al., 2021; Wescoe et al., 2014). We kept all unique aging sites from every submodel used for all further analyses, both for the 1 kb and the 10 kb dataset.

REFERENCES

1. Amat, F., Hontoria, F., O, R., Green, A., Sánchez, M., Figuerola, J., & Hortas, F. (2005). The American brine shrimp Artemia franciscana as an exotic invasive species in the Western Mediterranean. *Issues in Bioinvasion Science: EEI 2003: a Contribution to the Knowledge on Invasive Alien Species,* 37-47.
2. Beyter, D., Ingimundardottir, H., Oddsson, A., Eggertsson, H. P., Bjornsson, E., Jonsson, H., Atlason, B. A., Kristmundsdottir, S., Mehringer, S., Hardarson, M. T., Gudjonsson, S. A., Magnusdottir, D. N., Jonasdottir, A., Jonasdottir, A., Kristjansson, R. P., Sverrisson, S. T., Holley, G., Palsson, G., Stefansson, O. A., … Stefansson, K. (2021). Long-read sequencing of 3,622 Icelanders provides insight into the role of structural variants in human diseases and other traits. *Nature Genetics*, *53*(6), Article 6. https://doi.org/10.1038/s41588-021-00865-4
3. Canty, A., & Ripley, B. (2022). *boot: Bootstrap R (S-Plus) Functions.* (1.3-28.1) [Computer software]. https://cran.r-project.org/web/packages/boot/index.html
4. Gleeson, J., Leger, A., Prawer, Y. D. J., Lane, T. A., Harrison, P. J., Haerty, W., & Clark, M. B. (2022). Accurate expression quantification from nanopore direct RNA sequencing with NanoCount. *Nucleic Acids Research*, *50*(4), e19. https://doi.org/10.1093/nar/gkab1129
5. Kamacı, H., Çoban, D., Suzer, C., Aksu, B., Saka, Ş., & Fırat, M. K. (2010). Exocrine Pancreas Development and Trypsin Expression in Cultured European Sea Bass (*Dicentrarchus labrax*) Larvae. *Turkish Journal of Fisheries and Aquatic Sciences*, *10*. https://doi.org/10.4194/trjfas.2010.0117
6. Maechler, M., Rousseeuw, P., Croux, C., Todorov, V., Ruckstuhl, A., Salibian-Barrera, M., Verbeke, T., Koller, M., Conceicao, E., L. T., & di Palma, M., A. (2023). *robustbase: Basic Robust Statistics R package* (0.99-0) [Computer software]. http://CRAN.R-project.org/package=robustbase
7. Oksanen, J., Simpson, G., Blanchet, F., Kindt, R., Legendre, P., Minchin, P., O'hara, R., Solymos, P., Stevens, M., & Szoecs, E. (2022). *Vegan: Community Ecology Package, R Package.* (2.6-4) [Computer software].
8. Tourancheau, A., Mead, E. A., Zhang, X.-S., & Fang, G. (2021). Discovering multiple types of DNA methylation from bacteria and microbiome using nanopore sequencing. *Nature Methods*, *18*(5), Article 5. https://doi.org/10.1038/s41592-021-01109-3
9. Venables, W. N., & Ripley, B. D. (2002). Generalized Linear Models. In W. N. Venables & B. D. Ripley (Eds.), *Modern Applied Statistics with S* (pp. 183–210). Springer. https://doi.org/10.1007/978-0-387-21706-2_7
10. Wescoe, Z. L., Schreiber, J., & Akeson, M. (2014). Nanopores Discriminate among Five C5-Cytosine Variants in DNA. *Journal of the American Chemical Society*, *136*(47), 16582–16587. https://doi.org/10.1021/ja508527b
11. Wobbrock, J., Findlater, L., Gergle, D., & Higgins, J. (2011). The Aligned Rank Transform for Nonparametric Factorial Analyses Using Only ANOVA Procedures. In *Conference on Human Factors in Computing Systems—Proceedings* (Vol. 2011, p. 146). https://doi.org/10.1145/1978942.1978963
12. Zhang, Y., Akdemir, A., Tremmel, G., Imoto, S., Miyano, S., Shibuya, T., & Yamaguchi, R. (2020). Nanopore basecalling from a perspective of instance segmentation. *BMC Bioinformatics*, *21*(3), 136. https://doi.org/10.1186/s12859-020-3459-0
